# Could Olympic Gels of Polystyrene be Produced by ARGET ATRP From Bifunctional Initiators?

**DOI:** 10.1002/marc.202400564

**Published:** 2024-09-10

**Authors:** Niccolò Braidi, Nicola Porcelli, Fabrizio Roncaglia, Adele Mucci, Francesco Tassinari

**Affiliations:** ^1^ Department of Chemical and Geological Sciences University of Modena and Reggio Emilia Via Campi 103 Modena 41125 Italy

**Keywords:** ARGET ATRP, kinetics (polym.), olympic gels, styrene

## Abstract

The kinetics of gelation in the Activators Regenerated by Electron Transfer Atom Transfer Radical Polymerization (ARGET ATRP) of styrene, using a bifunctional initiator and no crosslinking agents are investigated. By applying the method of moments, we develop a system of differential equations that accounts for the formation of polymer rings. The kinetic rate constants of this model are optimized on the experimentally determined kinetics, varying the reaction temperature and ethanol fraction. Subsequently, we explore how variations in the amounts of catalyst, initiator, and reducing agents affect the simulated equilibria of ARGET ATRP, the emergence of gelation, and the swelling properties of the resulting networks. These findings suggest that favoring ring formation enhances the gelation phenomenon, supporting the hypothesis that the networks formed under the reported reaction conditions are olympic gels.

## Graphical abstract

A kinetic model for the ARGET ATRP of styrene from bifunctional initiators shows that higher amounts of ethanol, catalyst, and reducing agents, along with lower temperatures and initiator amounts, enhance the molar fraction of cyclic polystyrene. These trends correlate with the experimental effects on gelation time, yield, and swelling properties, supporting the hypothesized formation of olympic gels.

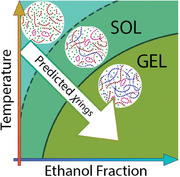

## 1 Introduction

Olympic gels (OGs) are polymer networks composed solely of concatenated polymer rings and thus are held together only by topological crosslinks.^[^ ^1^ ^]^ Direct observation of structures related to OGs was made in kinetoplast DNA, which contains thousands of concatenated DNA rings.^[^ ^2^, ^3^, ^4^, ^5^, ^6^ ^]^ Given their structure, OGs are expected to exhibit unusual viscoelastic behavior such as cooperative energy dissipation and rapid stress relaxation, yet only few theoretical investigations were reported.^[^ ^7^, ^8^ ^]^ Unfortunately, unlike other topological networks,^[^ ^6^, ^9^, ^10^ ^]^ the study of OGs properties has been limited by the significant synthetic challenge they present. Jacobson and Stockmayer first recognized that cyclization is an integral part of polycondensations.^[^ ^11^ ^]^ Although intuition suggests that the probability of cyclization decreases as polymer length or concentration increases, Stanford et al.,^[^ ^12^ ^]^ Gordon and Temple,^[^ ^13^, ^14^ ^]^ and Kricheldorf et al.^[^ ^15^ ^]^ later showed that polymer rings become the primary products at high conversions. As de Gennes first suggested,^[^ ^1^ ^]^ by favoring ring formation in a dilute regime and then gradually increasing the polymer concentration above the critical overlap concentration, it could be possible to synthesize OGs through the subsequent threading and concatenation of linear chains through already formed rings. This process would produce polymer rings with a significantly broadened molecular weight distribution due to simultaneous inter‐ and intra‐molecular polycondensations. Nonetheless, after removing the soluble fraction (linear chains and isolated rings), the remaining polymer would be a topological network composed solely of concatenated and dangling rings.

**Figure 1:**
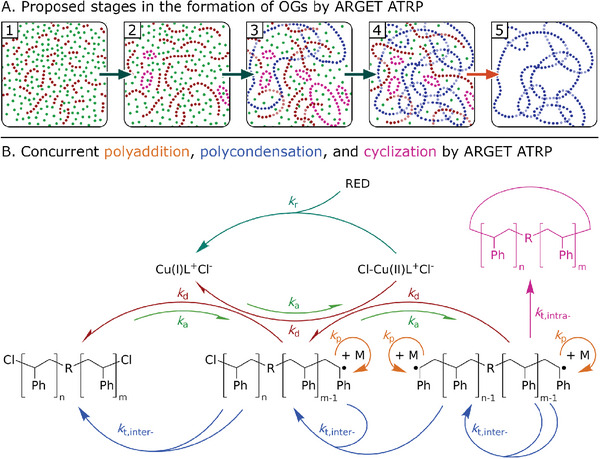
Proposed mechanism for the formation of OGs by ARGET ATRP from bifunctional initiators. A) Proposed stages of the reaction mixture: 1) a solution of linear telechelic chains (*red*) in styrene (*green*), 2) emergence of cyclic dead chains (*magenta*), 3) emergence of structures composed of concatenated rings (*blue*), 4) gelation of the reaction mixture via percolation of the topological network throughout the reaction volume, 5) OG retrieved after removing the soluble fraction. B) Schematic representation of the reactions needed to achieve concurrent polyaddition, polycondensation, and cyclization by ARGET ATRP: *activation* (*k*_a_), *deactivation* (*k*_d_), *reduction* (*k*_r_), *propagation* (*k*_p_), *intermolecular termination by coupling* (*k*_t,inter‐_), *intramolecular termination by coupling* (*k*_t,intra‐_). Note that we represent intermolecular terminations with two blue arrows converging at the coupling product. If two arrows start from a single substrate, it means two polymers with the same number of propagating chain‐ends react to form the indicated product.

Despite this and the fact that several chemistries could be employed to achieve simultaneous inter‐ and intra‐molecular polycondensations,^[^ ^6^, ^10^ ^]^ there are only a few reports of potential formation of OGs in the literature. Rigbi and Mark reacted telechelic α,ω‐dihydroxyl‐terminated polydimethylsiloxane with dimethylethoxysilane.^[^ ^16^ ^]^ Similarly, Hu et al. reacted heterotelechelic α‐vinyl,ω‐hydride polydimethylsiloxane with a Platinum catalyst.^[^ ^17^ ^]^ In both cases, the polycondensation products were rationalized as insoluble OGs mixed with linear chains and isolated rings. Interestingly, in the latter report, the networks exhibit stress‐strain responses consistent with sliding topological crosslinking points. Endo et al. reported the potential formation of OGs from the polymerization of 1,2‐dithiane.^[^ ^18^ ^]^ They found, via stress‐strain experiments, that the product was more flexible and could tolerate much higher deformations than the linear counterpart. The same synthetic approach was then applied to yield networks with carboxylic acid side groups or aromatic side groups.^[^ ^19^, ^20^ ^]^ To the best of our knowledge, aside from our seminal reports, no examples reportedly achieved OGs via radical polymerizations.^[^ ^21^, ^22^ ^]^ This approach would allow the use of industrially relevant monomers, potentially bearing functionalities that do not interfere with radical reactions, and more importantly, it would enable both batch production of OGs and the study of their properties. The advent of Atom Transfer Radical Polymerization (ATRP) has allowed the use of bifunctional initiators,^[^ ^23^, ^24^, ^25^ ^]^ which yield telechelic chains that can propagate at both ends while also allowing for simultaneous inter‐ and intramolecular polycondensations.^[^ ^26^, ^27^ ^]^ In the absence of monomer, telechelic polystyrene has been used to produce polymer rings through radical‐radical coupling.^[^ ^28^, ^29^ ^]^ In the presence of monomer, radical propagation allows the polymer concentration to cross the critical overlap concentration during the reaction. Consequently, one can envision that by favoring relatively high concentrations of radicals, it could be possible to favor the formation of polymer rings in a dilute regime and progressively concatenate them as the total polymer concentration increases while the concentration of active polymers (telechelic chains) remains low enough to favor cyclization over intermolecular coupling (**Figure** **1**A).

While studying the Activators Regenerated by Electron Transfer (ARGET) ATRP of styrene from bifunctional initiators, our group observed that employing sufficiently high amounts of reducing agents (RED) effectively forces the propagating telechelic chain to undergo termination by coupling.^[^ ^21^ ^]^ This observed superimposition of polyaddition and polycondensation implies a progressive decrease in the number of chain‐ends as the polymer concentration increases. In a subsequent paper, we postulated that the networks formed under these specific reaction conditions could be rationalized as OGs.^[^ ^22^ ^]^ In that paper, we focused on disproving that the cause of gelation was the in situ formation of canonical branching points (such as *β*‐elimination or mid‐chain hydrogen abstraction). We also qualitatively explored the effects on the emergence of gelation by varying the amounts of catalyst, initiator, and reducing agents. We observed that no gelation occurred above 90 °C or when the ethanol fraction in the reaction mixture (*ϕ*_EtOH_) was lowered. These observations were consistent with the notion that disfavoring the formation of radicals effectively favors propagation over radical‐radical termination,^[^ ^30^ ^]^ thus disfavoring the formation of OGs. Although we initially interpreted these results based on presumed effects on the Cu(I)/Cu(II) ratio, this interpretation lacked a systematic analysis of how these variations influence the process and the complex equilibria of ARGET ATRP (Figure 1B). Thus, in this present paper, we first conducted kinetic studies under different reaction conditions. Then, by developing a simple model that accounts for the formation of polymer rings, we evaluated how the explored variations affect the simulated equilibria of ARGET ATRP, the emergence of gelation, and the swelling properties of the obtained gels. The aim was to test the proposed hypothesis, namely that favoring ring formation enhances the gelation phenomenon.

## 2 Results and Discussion

**Figure 2:**
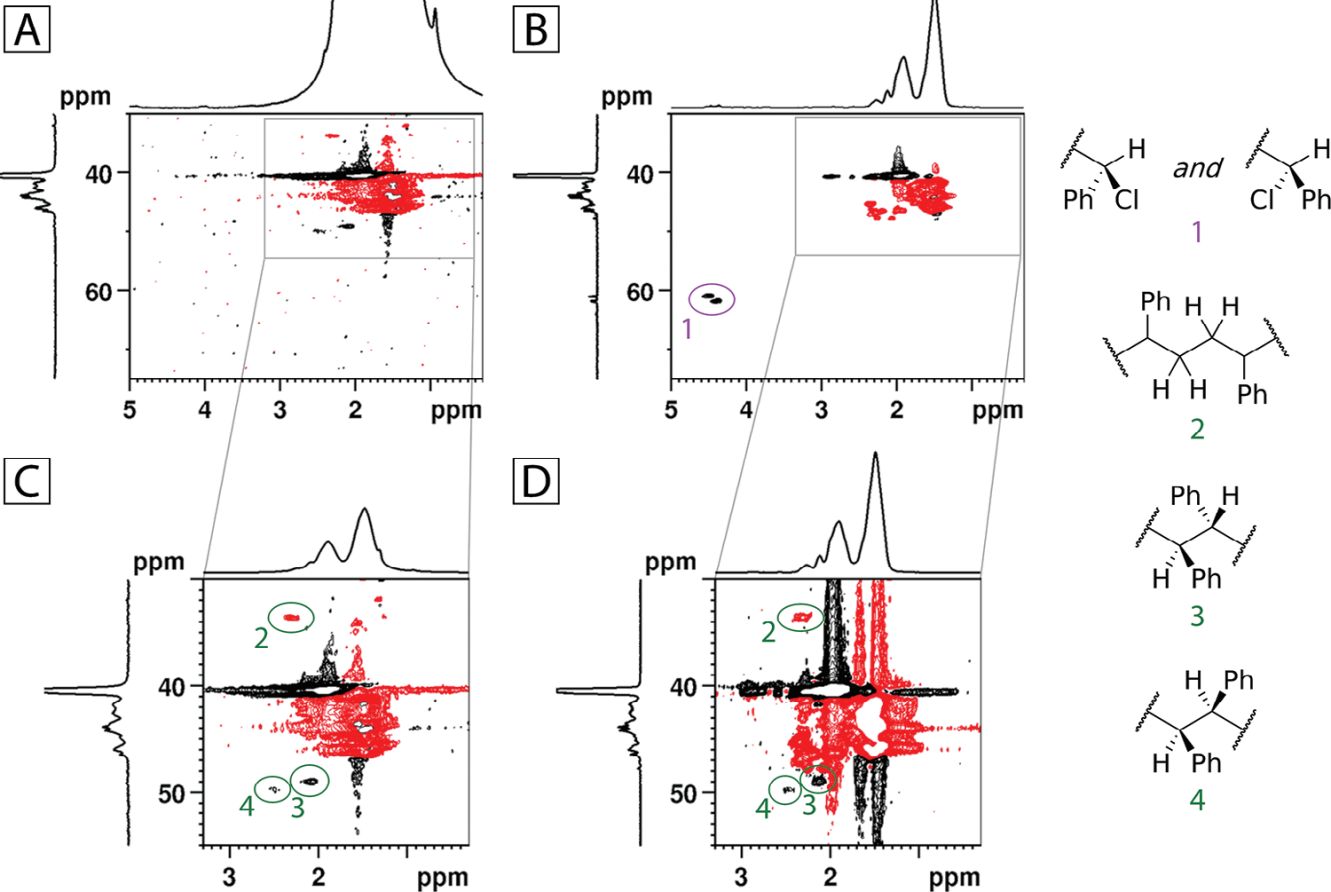
HSQCed spectrum of the polystyrene network after Soxhlet extraction with THF. A) and of a chloro‐terminated telechelic polystyrene B). The former has been obtained at the reaction conditions: [styrene]_0_:[α,α‐dichlorotoluene]_0_:[CuCl_2_/TPMA]_0_:[H_2_AA]_0_:[Na_2_CO_3_]_0_ = 100:1.06:0.05:0.5:1.5 mol% with respect to styrene, *V*_styrene_:*V*_EtOAc_:*V*_EtOH_ = 3:3:1 mL, *T* = 70 °C, *time* = 18 h, while the latter was obtained under the same reaction conditions except for: [H_2_AA]_0_:[Na_2_CO_3_]_0_ = 0:0.5 mol%, *T* = 100 °C, *time* = 5.5 h. C and D are magnifications of A and B, respectively. On the right, we have reported the protons (and related structures) attributed to the signals highlighted in the HSQCed spectra.

Under certain reaction conditions, the ARGET ATRP of styrene using bifunctional initiators (e.g., ethyl 2,2‐dichloropropanoate, EDCP) can result in gelation. Specifically, we encountered the anomalous phenomenon when using CuCl_2_/tris(2‐pyridylmethyl)amine (CuCl_2_/TPMA) as the catalyst, ascorbic acid and sodium carbonate as the reducing pair (H_2_AA:Na_2_CO_3_), and a solution of ethyl acetate and ethanol as the solvent (EtOAc:EtOH). For example, under the reaction conditions: [styrene]_0_:[EDCP]_0_:[CuCl_2_/TPMA]_0_:[H_2_AA]_0_:[Na_2_CO_3_]_0_ = 100:1.06:0.05:0.5:1.5 mol% with respect to styrene, *V*_styrene_:*V*_EtOAc_:*V*_EtOH_ = 3:3:1 mL, *T* = 70 °C, we reported how the polymer functionality (molar fraction of chlorinated chain‐ends) decreases until an insoluble network swollen with unreacted monomer and solvents is obtained.^[^ ^22^ ^]^ From the hypothesis formulated in the present introduction, if this gel is washed with a good solvent for polystyrene (e.g., tetrahydrofuran, THF), the NMR spectrum should show no signal attributable to the chlorinated chain‐ends. This is because only rings (either concatenated or dangling) should be part of the network, and the remaining linear chains and isolated rings should be washed away as a THF‐soluble fraction. To test this, we conducted a reaction under the conditions defined above, using α,α‐dichlorotoluene instead of EDCP as the initiator. The product was then extracted with a Soxhlet apparatus for 6 hours and dried until constant weight before being analyzed by NMR. The HSQCed spectrum of the network is shown in **Figure** **2**A, and as observed, there are no correlations around 4.5/62 ppm. For comparison, Figure 2B shows the HSQCed spectrum of a telechelic polystyrene obtained under different ARGET ATRP conditions, where radical‐radical terminations are strongly suppressed, and the chain‐end correlation is visible. In the magnified area of the first spectrum (Figure 2C), a CH signal at 2.05/49 ppm and a lower CH_2_ signal at 2.26/33.6 ppm, as well as an even lower one at 2.46/49.8 ppm, are visible. The chemical shifts of the two CH signals are in good agreement with head‐to‐head junctions (which differ in the relative positions of the two phenyl groups), and the CH_2_ shift corresponds to tail‐to‐tail junctions.^[^ ^31^ ^]^ Similarly, in the magnified area of the second spectrum (Figure 2D), the same set of signals is recognizable in the aliphatic portion of the spectrum. Crucially, the ratio of the sum of the areas of CH‐CH to the area of CH_2_‐CH_2_ is 0.9: 1 for the chlorinated telechelic, while in the network, the ratio is 1.5: 0.8. Thus, the HSQCed spectrum of the washed network is consistent with what would be expected from an olympic polystyrene, concatenated by radical‐radical coupling.

Following this, we wanted to test the proposed hypothesis by developing a model of the ARGET ATRP of styrene from bifunctional initiators that accounts for ring formation. To do so, we began by establishing a set of elementary reactions to represent the equilibria involved during the reaction (Figure 1B), these are: *activation*, *deactivation*, *reduction*, *propagation*, *inter‐* and *intra‐molecular couplings* (Equation (1), (2), (3), (4), (5), (6)). In the notation used, for a given polymer (P_n_^σ,j^), *n* represents the number of repeating units, *σ* the number of chain‐ends, and *j* (a subset of *σ*) the number of radical chain‐ends.

(1) $$activation,{\;\;P}_{n}^{\sigma ,j}+\mathrm{Cu}\left(I\right)L\;\overset{k_{a}}{\to }P_{n}^{\sigma ,j+1}+\mathrm{XCu}\left(\mathrm{II}\right)L\;\;\forall \;j<\sigma$$

(2) $$deactivation,{\;\;P}_{n}^{\sigma ,j}+\mathrm{XCu}\left(\mathrm{II}\right)L\;\overset{k_{d}}{\to }P_{n}^{\sigma ,j-1}+\mathrm{Cu}\left(I\right)L\;\;\forall \;j\geq 1$$

(3) $$reduction,\;\;\mathrm{RED}+\mathrm{XCu}\left(\mathrm{II}\right)L\overset{k_{r}}{\to }\mathrm{Cu}\left(I\right)L+\mathrm{OX}$$

(4) $$propagation,{\;\;P}_{n}^{\sigma ,j}+M\;\overset{k_{p}}{\to }P_{n+1}^{\sigma ,j}\;\;\forall \;j\geq 1$$

(5) $$intermol.\;coupling,{\;\;P}_{n_{A}}^{\sigma_{A},j_{A}}+P_{n_{B}}^{\sigma_{B},j_{B}}\overset{k_{t}}{\to }P_{n_{A}+n_{B}}^{\sigma_{A}+\sigma_{B}-2,j_{A}+j_{B}-2\;\;\;}\forall \;j\geq 1$$

(6) $$intramol.\;coupling,\;\;P_{n}^{\sigma ,j}\overset{k_{t}}{\to }P_{n}^{\sigma -2,j-2}\;\;\;\;\forall \;j\geq 2$$

We assume that these reactions represent a pseudo‐first‐order regime, with respect to monomer conversion, given the following approximations. First, we assume that partitioning between polymer and catalytic system does not occur, although the polymerization system is heterogeneous due to the use of a poorly soluble reducing pair in the reaction mixture. Second, we did not include a mode of inhibition of the catalytic system, despite our previous reports indicating that the use of Na_2_CO_3_ to promote reduction can cause this undesired reaction.^[^ ^32^, ^33^ ^]^ Third, we assumed a single mode of reduction since we maintained a constant ratio between H_2_AA and Na_2_CO_3_ throughout this study, thus representing them under the generic term RED, despite our previous reports indicating that this system is capable of different modes of reduction.^[^ ^33^, ^34^ ^]^ Note also that the oxidation products (grouped under the generic term OX, Equation 3) were not considered in the model. Fourthly, we imposed identical reactivity of the bifunctional initiator and the chain‐ends, representing the former as a telechelic chain without repeating units (*n* = 0), despite the significant difference in kinetic rate constant of activation between them.^[^ ^35^ ^]^ Last, we included only radical‐radical termination reactions by coupling, as in the ATRP of styrene this mode of termination is much more frequent than disproportionation.^[^ ^36^ ^]^ From these elementary reactions, we then applied the method of moments^[^ ^37^ ^]^ to derive the system of differential equations representing the equilibria at play (Equation S1–S16, Supporting Information). These equations rely upon the kinetic rate constants extrapolated from literature (*k*_p_(*T*),^[^ ^38^ ^]^ *k*_t_(*T*),^[^ ^39^, ^40^ ^]^ and *k*_d_(*T*),^[^ ^35^, ^41^ ^]^ row 1–3, Table S1, Supporting Information respectively), as well as those we need to optimize from experimental data, *k*_a_(*T*, *ϕ*_EtOH_) and *k*_r_(*T*, *ϕ*_EtOH_).

**Figure 3:**
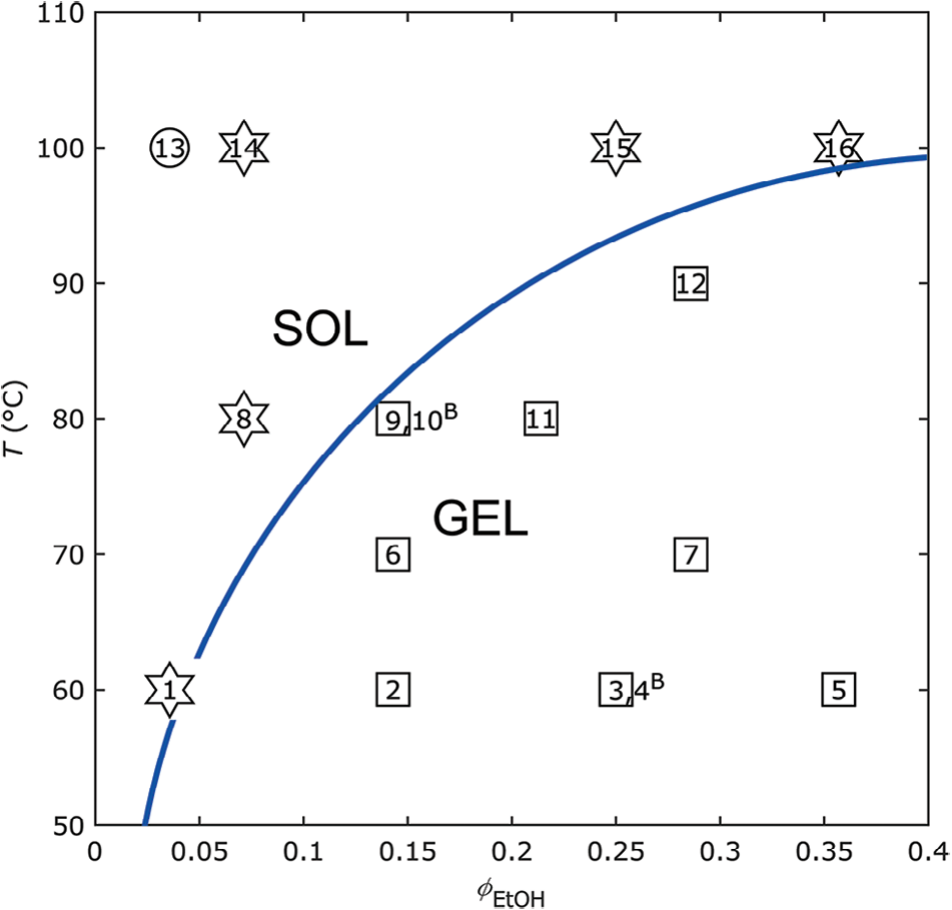
Representation of the reaction conditions reported in Table S2 (Supporting Information). Within 18 hours of reaction time, these reaction conditions yielded linear polymers (*circles*), branched structures (*stars*), or crosslinked products (*squares*). The continuous line serves as a visual guide to observe the emergence of macroscopic gelation with a decrease in *T* and/or an increase in *ϕ*_EtOH_. Common reaction conditions: [styrene]_0_:[EDCP]_0_:[CuCl_2_/TPMA]_0_:[H_2_AA]_0_:[Na_2_CO_3_]_0_ = 100:1.06:0.0125:0.5:1.5 mol% with respect to styrene, and *V*_styrene_:(*V*_EtOAc_ + *V*_EtOH_) = 3:4 mL, *time* = 18 h. Reactions 4^B^ and 10^B^ were conducted under the same conditions as reactions 3 and 9, respectively, but with [H_2_AA]_0_: [Na_2_CO_3_]_0_ = 0.125:0.375 mol%. Consequently, they are outside the experimental design defined by variations in *T* and *ϕ*_EtOH_. Note that both produced linear polymers.

To determine *k*_a_(*T*, *ϕ*_EtOH_) and *k*_r_(*T*, *ϕ*_EtOH_), we started by defining the experimental space identified by the experimental variables of interest (temperature and ethanol fraction, *T* and *ϕ*_EtOH_), as well as those held constant: [styrene]_0_:[EDCP]_0_:[CuCl_2_/TPMA]_0_:[H_2_AA]_0_: [Na_2_CO_3_]_0_ = 100:1.06:0.0125:0.5:1.5 mol% with respect to styrene, and *V*_styrene_:(*V*_EtOAc_ + *V*_EtOH_) = 3:4 mL. We deemed this experimental space to be especially fitting for defining the aforesaid kinetic rate constants because it encapsulates the emergence of gelation. As illustrated in **Figure** **3**, which plots the macromolecular architectures resulting from the experimental conditions reported in Table S2 (Supporting Information), an increase in *ϕ*_EtOH_ favors the emergence of gelation while an increase in *T* causes the disappearance of the phenomenon. It is worth noting that in most reaction conditions that do not produce insoluble gels, we detected branched structures by analyzing the soluble products through the coupled GPC‐Visco‐MALLS technique.^[^ ^21^, ^22^ ^]^ In fact, the only reaction condition that did not lead to gelation nor branched structures was found at high *T* (100 °C) and low *ϕ*_EtOH_ (0.0357 v/v), entry 13, Table S2 (Supporting Information). From this, we choose a full factorial design^[^ ^42^ ^]^ over *T* and *ϕ*_EtOH_, identified by: *T* ranging from 60 to 100 °C and *ϕ*_EtOH_ ranging from 0.0375 to 0.25 v/v. Then, we conducted the kinetics of the reaction conditions at the vertices of the design (entry 1,3,13, and 15, Table S2, Supporting Information). These were performed by carrying out each reaction condition for different reaction times to determine the evolution of conversion (*p*), number average molecular weight (*M*_n_), the percent difference between *M*_n_ and its theoretical value (Δ*M*_n_), and dispersity (*Ð*). The aim was to sample at least four points in the initial part of the reaction, which corresponds to a pseudo‐first‐order kinetic regime with respect to monomer conversion. The resulting kinetics were reported in Tables S3–S6 (Supporting Information) and plotted in Figures S1–S4 (Supporting Information), respectively.

**Figure 4:**
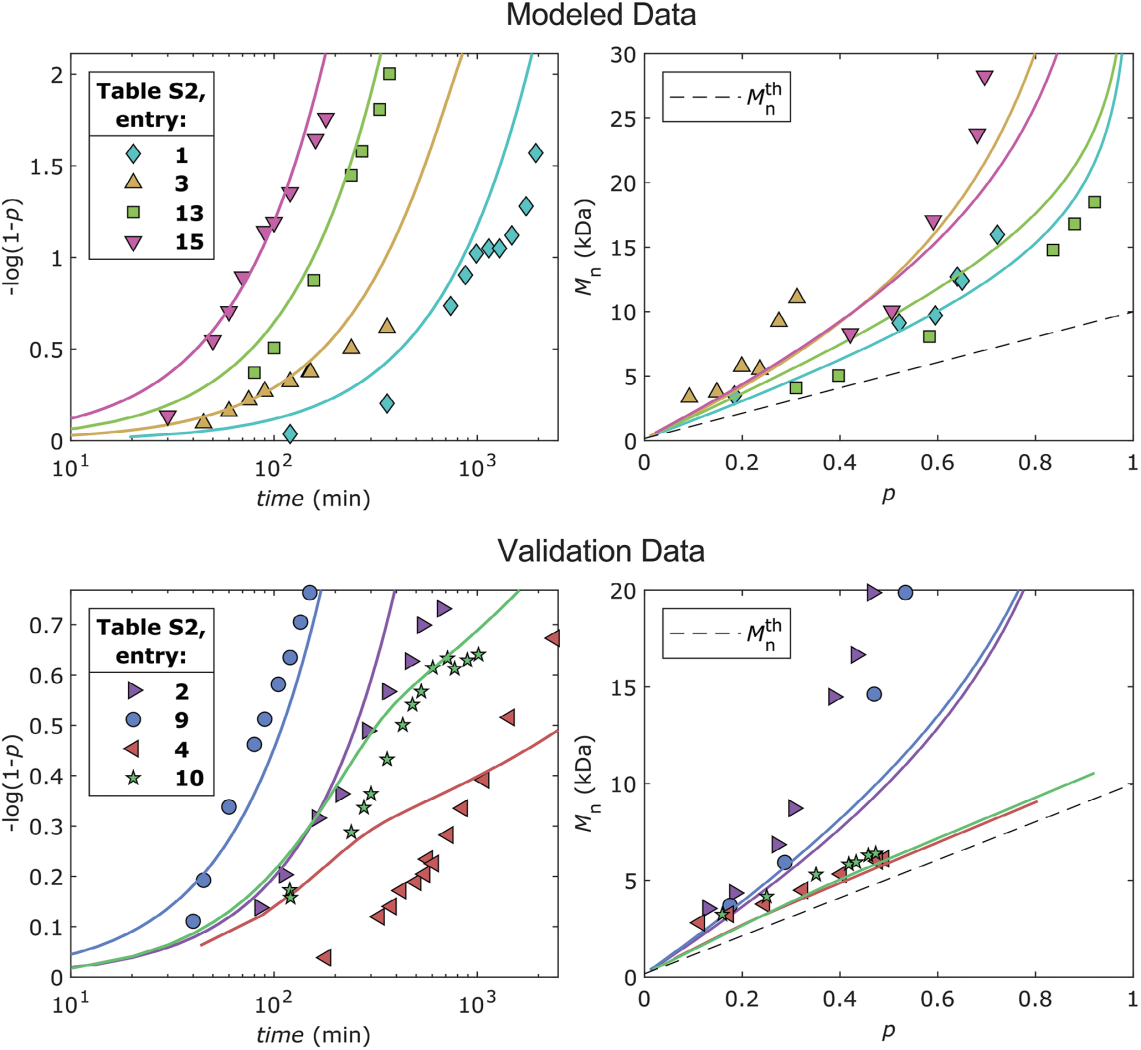
Kinetic evolution of selected reaction conditions reported in Table S2 (Supporting Information), experimental (*points*) against simulated results (*lines*). *Upper plots*, Modeled Data employed to optimize the kinetic rate constants of activation and regeneration. These kinetics were conducted at the same reaction conditions but with *T* (°C):*ϕ*_EtOH_ (v/v) = **1**) 60:0.0357, **3**) 60:0.25, **13**) 100:0.0357, **15**) 100:0.25. *Lower plots*, Validation Data employed to validate the developed model. These kinetics were conducted at the same reaction conditions but with [H_2_AA]_0_ (mol%):[Na_2_CO_3_]_0_ (mol%):*T* (°C):*ϕ*_EtOH_ (v/v) = **2**) 0.5:1.5:60:0.143, **9**) 0.5:1.5:80:0.143, **4**) 0.125:0.375:60:0.25, **10**) 0.125:0.375:80:0.143. *Left plots*, –log(1–*p*) versus time. *Right plots*, *M*_n_ versus *p* against *M*_n_^th^ versus *p* (dashed line). The individual kinetic plots, with time expressed in a linear scale, superimposed to the corresponding model predictions, have been reported in Figure S9 (Supporting Information).

By then combining these experimental data and the system of differential equations previously derived, we simultaneously optimized the values of *k*_a_(*T*, *ϕ*_EtOH_) and *k*_r_(*T*, *ϕ*_EtOH_). This was done by introducing a scalar entity, the L1 Norm calculated between the computed matrix of *p* and *M*_n_ and the experimentally determined one, which the optimization tool (particle swarm in MATLAB) then minimizes by varying the optimization constants (*c*_1_‐*c*_4_). The resulting *k*_a_(*T*, *ϕ*_EtOH_) and *k*_r_(*T*, *ϕ*_EtOH_) were reported as row 4 and 5, Table S1 (Supporting Information), respectively. As a first check of these results, we calculated K_ATRP_ (as the ratio *k*_a_/*k*_d_)^[^ ^30^, ^43^ ^]^ and found values ranging from 6.1e‐8 to 1.6e‐7 which are not dissimilar to the K_ATRP_ = 8.6e‐7 of α‐chloroethylbenzene with CuCl_2_/TPMA, in acetonitrile, previously reported by Tang and coworkers.^[^ ^35^ ^]^ As a second check, we observed that the developed model accurately represents the experimental points used for its optimization (Modeled Data, **Figure** **4**). Within the pseudo‐first‐order kinetic regime, the continuous line in –log(1–*p*) versus *time* (resulting from the model) closely approximates the experimental points. At longer reaction times, the model fails to capture the observed decrease in polymerization rate which we previously attributed to the inhibition of the catalyst.^[^ ^32^, ^33^ ^]^ Note that in Figure 4 we have plotted the data by expressing time in logarithmic scale so that both fast and slow kinetics can be appreciated at once, in Figure S9 (Supporting Information) we have instead plotted each kinetic evolution individually so that the linearity of the initial polymerization regime can be appreciated better. Regarding *M*_n_, the model accurately predicts its pseudo‐exponential increase with increasing *p*, although the drifts between experimental points and simulated lines are noticeable. Subsequently, we conducted four additional kinetics to adequately validate the model and test its limits. These included points both within the experimental space of the full factorial design (entry 2 and 9, Table S2, Supporting Information), as they were conducted at intermediate values of *T* and/or *ϕ*_EtOH_, and outside of it (entry 4 and 10, Table S2, Supporting Information), as they were conducted with quantities of reducing agents decreased by fourfold. As depicted in the Validation Data, Figure 4, the model exhibits lower accuracy in predicting points inside the experimental space compared to its vertices. Since the applied method uses only four optimization constants, we attributed such deviations to a non‐linear dependance on *T* and *ϕ*_EtOH_, which had to be expected given the complexity of the system. As for points outside the experimental space, the accuracy of –log(1–*p*) versus *time* significantly worsens, although the model correctly predicts that the radical‐radical termination reactions are greatly suppressed as the quantity of reducing agents is decreased fourfold. In fact, entry 4 and 10, Table S2 (Supporting Information), did not lead to gelation nor branched structures.

**Figure 5:**
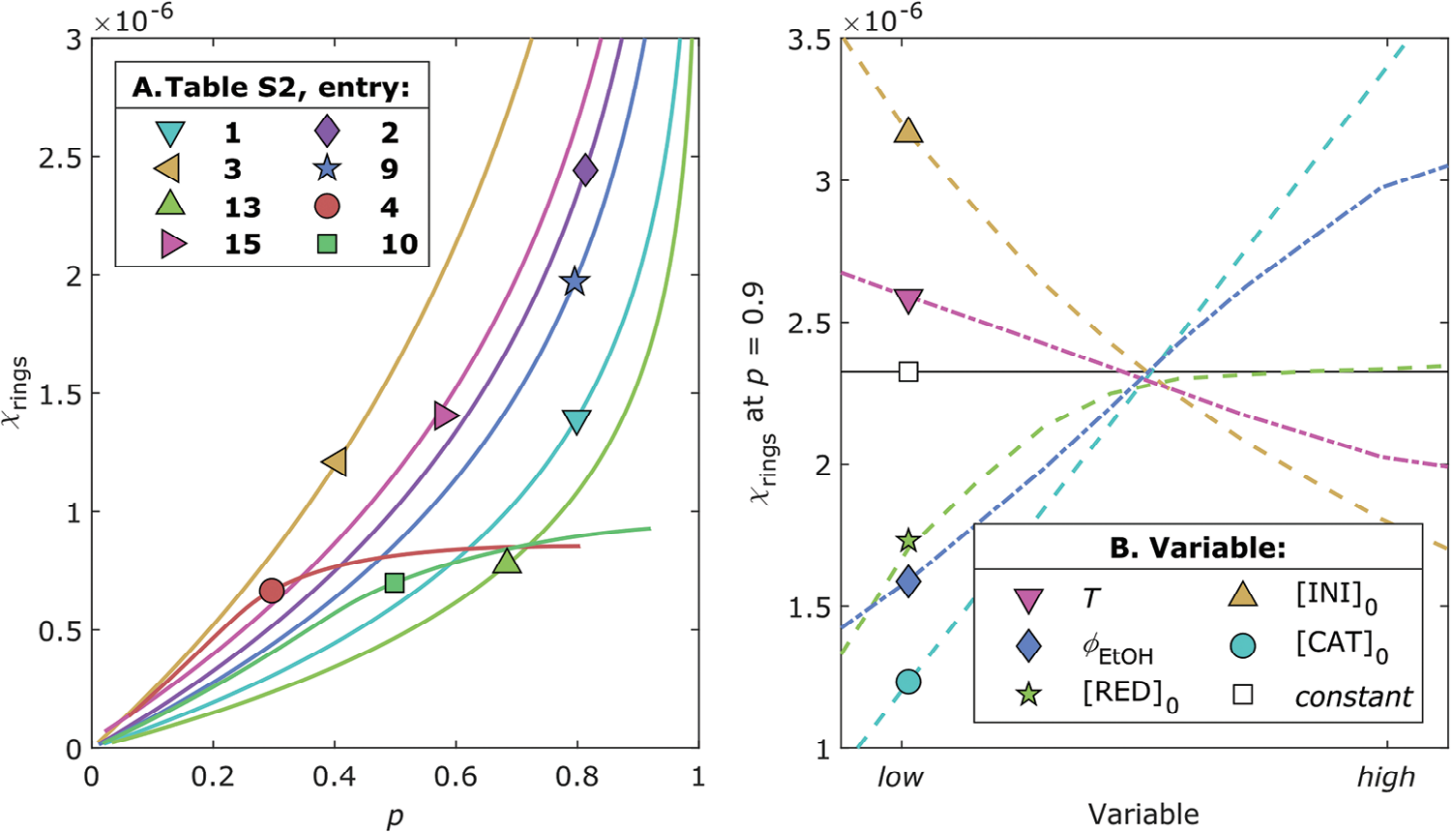
**A**) Evolution of the simulated *χ*_rings_ as a function of *p* (calculated with Equation S17, Supporting Information). The reaction conditions, reported in Table S2 (Supporting Information), differ by *T* (°C):*ϕ*_EtOH_ (v/v) as follows: 1) 60:0.0357, **3**) 60:0.25, **13**) 100:0.0357, **15**) 100:0.25, **2**) 60:0.143, **9**) 80:0.143, **4**) 60:0.25, **10**) 80:0.143. Additionally, reactions **4** and **10** were simulated with [H_2_AA]_0_ (mol%):[Na_2_CO_3_]_0_ (mol%) = 0.125:0.375 instead of 0.5:1.5 as the others. **B**) Simulated *χ*_rings_ values at *p* = 0.9 as a function of one changing variable at a time. The explored ranges for each variable are: *T* from 60 to 100 °C, *ϕ*_EtOH_ from 0.0357 to 0.25 v/v, [RED]_0_ from 0.125:0.375 to 1:3 mol%, [INI]_0_ from 1 to 2 mol%, [CAT]_0_ from 0.00625 to 0.025 mol%, thus simulating the experimental space explored in Table S11 (Supporting Information).

Given these results, we then tested the hypothesis proposed in the introduction. To do so, we extrapolated from the model the molar fraction of polymer rings (*χ*_rings_), as a function of *p*, *T*, and *ϕ*_EtOH_, which is calculated as the ratio between the zero‐th moment of polymer rings and the sum of all zero‐th moments of polymers (Equation S17, Supporting Information). First, **Figure** **5**A shows that the model predicts a pseudo‐exponential increase in *χ*_rings_ with increasing *p*. This observation is in accordance with what is known about irreversible polycondensations. In fact, it has been theorized that, at *p* = 1, an ideal irreversible polycondensation results in a polymer composed of *χ*_rings_ = 1, without linear chains.^[^ ^15^ ^]^ The second observation we can draw is that, at a given *p*, a higher *T* disfavors the formation of rings, while conversely, a higher *ϕ*_EtOH_ favors it. This result is in line with what we observed in Figure 3, where the macroscopic gelation emerges by lowering *T* and increasing *ϕ*_EtOH_, while gradually disappearing in the opposite direction.

To further test our hypothesis, we subsequently explored the model predictions in response to variations in the quantities of reagents instead of *T* and *ϕ*_EtOH_. Specifically, we varied the initial amount of catalyst ([CAT]_0_) from 0.00625 to 0.025 mol% with respect to [styrene]_0_, initiator ([INI]_0_) from 1 to 2 mol%, and reducing agents ([RED]_0_) from 0.5:1.5 to 1:3 mol%, while keeping constant: [styrene]_0_ = 100 mol%, *T* = 70 °C, *ϕ*_EtOH_ = 0.143 v/v, and *time* = 1080 min. As we saw in Figure 5A, an increase in [RED]_0_ results in a significant increase of *χ*_rings_(*p*): compare entry 3 versus 4 and 9 versus 10, Table S2 (Supporting Information). This leads to diminishing returns, and the model barely predicts any difference from the further increase in [RED]_0_ in the second experimental space (Figure 5B). Although the influence of [RED]_0_ was expected, the effects of [CAT]_0_ and [INI]_0_ are less immediate. As observed from the model's prediction, an increase in [CAT]_0_ leads to an increase in *χ*_rings_(*p* = 0.9), whereas an increase in [INI]_0_ results in a decrease in it (Figure 5B). To validate these predicted trends, we conducted a series of polymerizations within the second experimental space (Table S11, Supporting Information). Since the performed series of reaction conditions reliably leads to gelation, here we focused on the effects that the variations in the reagents quantities exert on the properties of the obtained crosslinked products, instead of the kinetics. These properties, which will serve as response factors, were determined following the procedure detailed in the experimental section. Briefly, by monitoring the reaction through time‐lapse we determine the gelation time (*t*_GP_) as the instant at which the stirring bar stops. The product is then retrieved from the Schlenk tube, soaked in methanol, dried and weighted to determine the *yield*. It is then portioned in small slices, soaked again in methanol for a week and dried, and finally swelled in toluene to determine the swelling degree (*Q*) and gel fraction (*%G*). From the boxplots of the individual response factors, we detect no outliers (Figure S10, Supporting Information). Additionally, from the correlation matrix plot, we find no significant correlation among the experimental variables (Figure S11, Supporting Information). These two observations allowed us to perform stepwise regression on Table S11 (Supporting Information), to correlate the response factors with the explored variables, without fear of confounding the main effects.^[^ ^42^ ^]^ In Figure S12 (Supporting Information), we reported the diagnostic plots of the residuals of the four linear models. Observing the histogram plot along with the normal probability plot of the residuals allows us to conclude that the residuals are normally distributed in all four models.

As for the plots of fitted values versus residuals, which should also display a normal distribution around zero, we observe that only the plot for the model of *t*_GP_ exhibits a non‐random pattern (Figure S12, Supporting Information). The observed U‐shaped distribution might suggest that a significant quadratic term is not included in the linear model. We considered it inappropriate to include quadratic terms in the stepwise modeling due to the relatively low number of observations collected.^[^ ^42^ ^]^ Since the diagnostic plots of the models do not indicate outliers, we can confidently comment the results of the stepwise linear regression, reported in **Table** **1**. The linear model describing *t*_GP_ includes only predictors related to [INI]_0_ and [RED]_0_, which have a similar magnitude but opposite signs. That is, an increase in the initiator quantity enhances the reaction time needed for gelation to occur, while an increase in the reducing agents quantity accelerates the onset of gelation. The linear model describing yield includes all three predictors, even though the magnitude of the [CAT]_0_‐related predictor is an order of magnitude lower than the other two. For this response factor, also the interaction terms related to [INI]_0_ (*b*_13_ and *b*_21_) are considered significant. The terms predicting the behavior of *Q* are instead more similar in magnitude to each other. In this case, the only term judged non‐significant at the imposed confidence level is the interaction term of [CAT]_0_·[INI]_0_. An increase in the quantities of [CAT]_0_ and [RED]_0_ corresponds to an increase in the *Q* of the product, while an increase in [INI]_0_ corresponds to a decrease in it. Based on the inverse correlation between *Q* and *%G* (as shown in the correlation matrix, Figure S11, Supporting Information), it should be expected that the same variations correspond to a decrease in *%G*. Indeed, we observed significant and negative values for *b*_2_ and *b*_3_, while *b*_1_ is positive. In this case, however, no interaction term was found to be significant. Given this, if a higher *χ*_rings_/*p* ratio leads to an anticipation of the gelation phenomenon, as it anticipates the conversion at which the crosslinked structure has enough topological crosslinks to percolate through the reaction volume, then the significant effects observed from the stepwise linear modeling are in good agreement with the simulated effects. In other words, by employing relatively higher [RED]_0_ and/or lower [INI]_0_ (variations that enhance the *χ*_rings_/*p* ratio, Figure 5B), we anticipate the onset of the gelation (lower *t*_GP_). Since gelation dramatically slows all reactions due to a sharp increase in viscosity, it stands to reason that by anticipating its onset, lower conversions are achieved. In fact, *t*_GP_ is directly correlated with the *yield* (Figure S11, Supporting Information), and similarly, *b*_1_ and *b*_3_ respectively share the same sign in both linear models. It is noteworthy that the explored variations also influence the properties of the gelled product (*Q* and *%G*). This can again be attributed to the time needed for gelation to occur, since faster gelation means that less monomers have been incorporated into the crosslinked product by propagation and entanglement between chains. Thus, the same volume is occupied by fewer repeating units that constitute the crosslinked product. Consequently, the products swell more (higher *Q*) and show a lower *%G*. In summary, our tests have shown a strong correlation between the fraction of produced rings (as simulated using a simple system of differential equations) and the emergence of the gelation phenomena. This correlation is evident both from a kinetic perspective (changes in *T* and *ϕ*_EtOH_) and from a process and product properties perspective (changes in [CAT]_0_, [INI]_0_, and [RED]_0_). Consequently, we believe these findings support the hypothesis that the formation of an OG underpins the gelation phenomena observed. However, the model predicts *χ*_rings_ values on the order of 10^–6^. It is difficult to accurately determine whether this value is high enough to justify macroscopic gelation, particularly without experimental data to estimate the number of chains that a single ring could thread (and thus the number of topological crosslinks it produces). Moreover, if we assume that the reaction occurs in a homogeneous solution – as implied by the system of differential equations used – the predicted *χ*_rings_ values are certainly overestimated because: i) the reported model did not account for viscosity‐dependent changes in the kinetic rate constants of termination, which are known to decrease more than other kinetic constants as the reaction progresses,^[^ ^44^ ^]^ ii) we imposed an intra‐molecular termination kinetic rate constant equal to the inter‐molecular kinetic rate constant, without accounting for the decrease in the probability of ring formation as the polymer length increases. Crucially, the polymerization system in which this gelation is encountered is nonetheless heterogeneous. It contains barely soluble reducing agents, a non‐solvent of polystyrene (ethanol), and one of the reducing agents releases water, another non‐solvent for polystyrene. This makes it plausible to expect a partition between the polymeric chains and the catalytic system during the reaction which might promote termination reactions over propagation.^[^ ^45^, ^46^ ^]^ In conclusion, considering the reported results, we argue that the formation of OGs by ARGET ATRP is feasible, provided that radical‐radical terminations reactions (by coupling) are favored over propagation. However, as we have just discussed, future work is needed to clarify the role that heterogeneity exerts on the system and whether it is possible to obtain OGs from ATRP in a homogeneous system.

**Table 1:** Intercept (*b*_0_), predictors (*b*_1_‐*b*_3_), and interaction terms (*b*_13_‐*b*_32_) of the four models describing how [INI]_0_, [CAT]_0_, and [RED]_0_ influence *t*_GP_, *yield*, and the gel properties (*Q* and *%G*).^a^ ^)^

Coefficients	*t*_GP_ [min]	*yield* [mg]	*Q* [v/v]	*%G* [%]
*b*_0_ (*intercept*)	+373.6	+2.89e+3	+22.2	+76.0
*b*_1_ ([INI]_0_)	+141.9	+944.4	−22.1	+8.26
*b*_2_ ([CAT]_0_)	n.s.	+93.7	+10.7	−13.2
*b*_3_ ([RED]_0_)	−112.0	−544.0	+11.3	−6.51
*b*_13_ ([INI]_0_·[RED]_0_)	n.s.	−248.83	+11.7	n.s.
*b*_21_ ([CAT]_0_·[INI]_0_)	n.s.	+174.2	n.s.	n.s.
*b*_32_ ([RED]_0_·[CAT]_0_)	n.s.	n.s.	−4.2	n.s.

^a)^ Coefficients defined by the four stepwise linear modeling. Terms are deemed non‐significant (n.s.) with an imposed confidence level of 95%.

## 3 Conclusion

In this work, we aimed to test the hypothesis that ring formation underlies the gelation in the ARGET ATRP of styrene using bifunctional initiators. To achieve this, we started by developing a theoretical model to represent the dynamic equilibria during the reaction. The kinetic rate constants of activation and reduction governing these equilibria were optimized based on experimental data. These were collected by performing kinetic analysis of reaction conditions stemming from a systematic variation in temperature and ethanol fraction, particularly in a range that exhibits the emergence of gelation. By extrapolating the simulated molar ratio of polymer rings, we found direct correlation between the experimental results and simulations. Specifically, where experiments showed the emergence of gelation at lower temperatures and higher ethanol fractions, simulations resulted in an increase in the molar fraction of rings at a given conversion. Similarly, we then applied the model to predict the effects of variations in the quantities of catalyst, initiator, and reducing agents. A series of reactions conducted over this second experimental space again confirmed that the predicted increase in the molar fraction of polymer rings enhances the gelation phenomenon. By adjusting the reagents quantities to favor ring formation (high quantities of catalyst and reducing agents, or a low quantity of initiator), we observed an earlier onset of gelation, characterized by decreased yield, higher degrees of swelling, and lower gel content. The limitations of this approach stem from the simplicity with which the model was derived. By describing a homogeneous polymerization – despite the inherently heterogeneous nature of the reported ARGET ATRP – the model predicts extremely low values of the molar fraction of ring polymers. Therefore, although these results support the proposed hypothesis, further investigations are necessary to determine the effects of the system heterogeneity on the formation of OGs by ATRP.

## Conflict of Interest

The authors declare no conflict of interest.

## Supporting information



Supporting Information

## Data Availability

The data that support the findings of this study are available in the supplementary material of this article.
