# Supplementary material for: Could Olympic Gels of Polystyrene be Produced by ARGET ATRP From Bifunctional Initiators?
Source: Macromol Rapid Commun. 2024 Sep 10;46(1):2400564. doi: 10.1002/marc.202400564 (PMC11713849; doi:10.1002/marc.202400564)
Supplement: Supplementary file 1 — Supporting Information [file MARC-46-2400564-s001.pdf]

Macromolecular Rapid Communications

Supporting Information

for *Macromol. Rapid Commun.*, DOI 10.1002/marc.202400564

Could Olympic Gels of Polystyrene be Produced by ARGET ATRP From Bifunctional Initiators?

Niccolò Braidi, Nicola Porcelli, Fabrizio Roncaglia, Adele Mucci and Francesco Tassinari**

Supporting Information

Could Olympic Gels of Polystyrene be Produced by ARGET ATRP from Bifunctional Initiators?

Niccolò Braidi,[1] Nicola Porcelli,[1] Fabrizio Roncaglia,[1] Adele Mucci,[1] and Francesco Tassinari[1]**

Department of Chemical and Geological Sciences, University of Modena and Reggio Emilia (Italy), Via Campi 103, 41125 Modena, Italy.

E-mail: niccolo.braidi2@unibo.it, francesco.tassinari@unimore.it

EXPERIMENTAL SECTION

Materials

The commercial reagents were used as received: styrene stabilized with 10-15 ppm of 4-tert-butylcatechol (supplied by Versalis (ENI) S.p.A., > 99.0%), copper (II) chloride (Riedel-de Hann, ≥ 97%), tris(2-pyridylmethyl)amine (TCI Europe, 98%), ascorbic acid (Merck, > 99.5%), sodium carbonate (Carlo Erba, ≥ 99.5%), ethanol (Merck, ≥99.8%), ethyl acetate (Merck, ≥ 99.5%), dichloromethane (Fisher Chemical, ≥ 99.8%), methanol (Chemlab, > 99%). Ethyl 2,2-dichloropropionate (EDCP) was prepared by esterification of ethanol with 2,2-dichloropropionyl chloride (the latter was obtained following ref.1).

ARGET ATRP

As a representative example: ascorbic acid (23.0 mg, 0.130 mmol) and sodium carbonate (41.5 mg, 0.392 mmol) were weighted and transferred into an oven-dried 25 mL Schlenk tube. Three vacuum/argon cycles were then performed. Under argon flow, the following reagents were added in order: styrene (3 mL, 26.1 mmol), ethyl acetate (2 mL), ethyl 2,2-dichloropropionate (47.2 mg, 0.276 mmol, as a solution in ethyl acetate, 1 mL), ethanol (0.94 mL), copper (II) chloride/tris(2-pyridylmethyl)amine (0.439/0.947 mg, 0.00163/0.00163 mmol, as a solution in ethanol, 62.5 µL). The Schlenk tube was then sealed under argon and immersed in a pre-heated oil bath (80 °C). After the desired reaction time, if the reaction mixture did not gel, dichloromethane (10 mL) was added to Schlenk tube and the product precipitated in a large excess of methanol (400 mL). To aid coagulation, a solution of HCl (2 mL, 5% w/V) was added. The precipitate was filtered through a filter funnel with sintered glassfilter (ROBU®, P4) and washed twice with methanol. After drying the filtrate, the conversion (p) and consequently the theoretical number average molar mass (M_n^{th}) as well as the percent difference between M_n and its theoretical value (ΔM_n) were determined gravimetrically.[2,3]

Where:

$$M_{\mathrm{n}}^{\mathrm{th}} = \frac{[\mathrm{styrene}]_0}{[\mathrm{initiator}]_0} \cdot p \cdot \mathrm{MW}_{\mathrm{styrene}} + \mathrm{MW}_{\mathrm{initiator}}$$

$$\Delta M_{\mathrm{n}} = \frac{M_{\mathrm{n}} - M_{\mathrm{n}}^{\mathrm{th}}}{M_{\mathrm{n}}^{\mathrm{th}}} \cdot 100$$

Otherwise, if the reaction mixture gelled, methanol (2 mL) is added to the Schlenk tube, and the product is recovered with the help of a spatula. The product is then immersed in methanol—given the partially open-shell topology of the crosslinked product, most of the reaction solvents and unreacted monomer are thus removed without removing the linear chains or isolated rings. The product is subsequently dried to estimate the yield. Next, the product is sliced into thin discs, which are washed again in methanol and then dried.

Gel Permeation Chromatography (GPC)

GPC was performed on a Water GPC system composed of a separation module: Waters Alliance 2695, columns: four Phenogel (Phenomenex) with size 300 x 7.6 mm with particle size 5 µm and porosities of 1e+6, 1e+5, 1e+4, and 1e+3 Å, differential refractometer detector: Water 2414, software: Empower 2. The system was calibrated with 20 narrowly distributed polystyrene standards with MW ranging from 1.3 to 7’000 kDa. Tetrahydrofuran HPLC-grade was used as the mobile phase (1 mL/min, sample concentration 2.5 mg/mL) with toluene as the internal standard. From the obtained MWDs we were able to derive the number and mass average molar masses (M_{n} and M_{w}, respectively) and dispersity as a consequence ($Đ = M_{\mathrm{w}}/M_{\mathrm{n}}$). The coupled technique GPC-Visco-MALLS (MALLS) analysis was performed using the same GPC system as above, additionally equipped with a MALLS DAWN EOS WYATT and Viscotek T50 A as light scattering and viscometer detectors. The sample concentration was 1.0 mg/mL.

Nuclear Magnetic Resonance (NMR)

NMR spectra were recorded with an AVANCE III HD 600 spectrometer (Bruker BioSpin GmbH, Rheinstetten, Germany) equipped with a CryoProbe BBO H&F 5 mm (operating at 600.13 and 150.90 MHz for ^{1}H and ^{13}C, respectively). The H,C-Heteronuclear Single Quantum Coherence edited (HSQCed) spectra were acquired on polystyrenes swelled/dispersed in $CDCl_3$ using a standard echo-antiecho phase-sensitive pulse sequence (hsqcedetgpsp.3, Bruker library) and the following relevant acquisition parameters: 1 s relaxation delay, 1.786 ms evolution time, 7 kHz spectral width in f2, 2k data points, 24 scans per increment for Olympic polystyrene and 16 scans for chloro-terminated telechelic polystyrene, 27 kHz spectral width in f1, and 512 increments for olympic and 400 increments for telechelic polystyrene.

Swelling tests

They were conducted in glass vials, on samples weighing 50-100 mg (w_{start}), to which toluene (40 mL) was added. After 24 h of swelling the sample was retrieved, weighted (w_{swollen}), and dried in a vacuum oven at 90 °C for 24 h (w_{dry}). This allows to determine both the swelling degree (Q) and the gel fraction ($\%G$) as follows:

$$Q = 1 + \left[\frac{\rho_{\mathrm{PS}}}{\rho_{\mathrm{toluene}}} \cdot \left(\frac{w_{\mathrm{swollen}}}{w_{\mathrm{dry}}} - 1\right)\right]$$

$$\%G = \frac{w_{\mathrm{dry}}}{w_{\mathrm{start}}} \cdot 100$$

DIFFERENTIAL EQUATIONS

Starting from the elementary reactions reported in the manuscript (**Equation 1**–**6**), we derived, using the method of moments, the system of differential equations that will serve as the model for the ARGET ATRP of styrene from bifunctional initiators. This was done following the procedure detailed by E. Mastan and S. Zhu.[4] Briefly, we first wrote the mass balances of the elementary reactions listed, both for the small molecules and for polymers with a generic number of monomers, *n*. It was essential to explicitly define all possible populations (having imposed *a priori* that in the system of interest *σ* can be equal to 0 or 2). For example, the mass balance of Cu(I)L, given **Equation 1**–**3**, is expressed as:

$$\frac{d[\mathrm{Cu(I)L}]}{dt} = -\,2\cdot \mathrm{k_a}\cdot[\mathrm{Cu(I)L}]\cdot\sum_{n\,=\,0}^{\infty}\left[\mathrm{P}_n^{2,0}\right] - \mathrm{k_a}\cdot[\mathrm{Cu(I)L}]\cdot\sum_{n\,=\,0}^{\infty}\left[\mathrm{P}_n^{2,1}\right] + \mathrm{k_d}\cdot[\mathrm{XCu(II)L}]\cdot\sum_{n\,=\,0}^{\infty}\left[\mathrm{P}_n^{2,1}\right] + 2\cdot\mathrm{k_d}\cdot[\mathrm{XCu(II)L}]\cdot\sum_{n\,=\,0}^{\infty}\left[\mathrm{P}_n^{2,2}\right] + \mathrm{k_r}\cdot[\mathrm{RED}]\cdot[\mathrm{XCu(II)L}]$$

After this, we derived the system of differential equations (**Equation S1**–**S17**) by applying the definition of the k-th moment of the polymer population with *σ* chain-ends and *j* (a subset of *σ*) number of activated chain-ends :

$$\mathrm{S}_{\mathrm{k}}^{\sigma,j} = \sum_{\mathrm{k}\,=\,0}^{\infty} n^{\mathrm{k}}\cdot\left[\mathrm{P}_n^{\sigma,j}\right]$$

Equation S1, $\frac{d[\mathrm{Cu(I)L}]}{dt} = -\,2\cdot\mathrm{k_a}\cdot S_0^{2,0}\cdot[\mathrm{Cu(I)L}] - \mathrm{k_a}\cdot\mathrm{S}_0^{2,1}\cdot[\mathrm{Cu(I)L}] + \mathrm{k_d}\cdot\mathrm{S}_0^{2,1}\cdot[\mathrm{XCu(II)L}] + 2\cdot\mathrm{k_d}\cdot\mathrm{S}_0^{2,2}\cdot[\mathrm{XCu(II)L}] + \mathrm{k_r}\cdot[\mathrm{RED}]\cdot[\mathrm{XCu(II)L}]$

Equation S2, $\frac{d[\mathrm{XCu(II)L}]}{dt} = +2\cdot\mathrm{k_a}\cdot\mathrm{S}_0^{2,0}\cdot[\mathrm{Cu(I)L}] + \mathrm{k_a}\cdot\mathrm{S}_0^{2,1}\cdot[\mathrm{Cu(I)L}] - \mathrm{k_d}\cdot\mathrm{S}_0^{2,1}\cdot[\mathrm{XCu(II)L}] - 2\cdot\mathrm{k_d}\cdot\mathrm{S}_0^{2,2}\cdot[\mathrm{XCu(II)L}] - \mathrm{k_r}\cdot[\mathrm{RED}]\cdot[\mathrm{XCu(II)L}]$

Equation S3, $\frac{d[\mathrm{RED}]}{dt} = -\mathrm{k_r}\cdot[\mathrm{RED}]\cdot[\mathrm{XCu(II)L}]$

Equation S4, $\frac{d[\mathrm{M}]}{dt} = -\mathrm{k_p}\cdot[\mathrm{M}]\cdot\mathrm{S}_0^{2,1} - 2\cdot\mathrm{k_p}\cdot[\mathrm{M}]\cdot\mathrm{S}_0^{2,2}$

Equation S5, $\frac{d[\mathrm{S}_0^{2,0}]}{dt} = -2\cdot\mathrm{k_a}\cdot\mathrm{S}_0^{2,0}\cdot[\mathrm{Cu(I)L}] + \mathrm{k_d}\cdot\mathrm{S}_0^{2,1}\cdot[\mathrm{XCu(II)L}] + \mathrm{k_t}\cdot\mathrm{S}_0^{2,1}\cdot\mathrm{S}_0^{2,1}$

Equation S6, $\frac{d[\mathrm{S}_1^{2,0}]}{dt} = -2 \cdot \mathrm{k_a} \cdot \mathrm{S}_1^{2,0} \cdot [\mathrm{Cu(I)L}] + \mathrm{k_d} \cdot \mathrm{S}_1^{2,1} \cdot [\mathrm{XCu(II)L}] + 2 \cdot \mathrm{k_t} \cdot \mathrm{S}_1^{2,1} \cdot \mathrm{S}_0^{2,1}$

Equation S7, $\frac{d[\mathrm{S}_2^{2,0}]}{dt} = -2 \cdot \mathrm{k_a} \cdot \mathrm{S}_2^{2,0} \cdot [\mathrm{Cu(I)L}] + \mathrm{k_d} \cdot \mathrm{S}_2^{2,1} \cdot [\mathrm{XCu(II)L}] + 2 \cdot \mathrm{k_t} \cdot \mathrm{S}_1^{2,1} \cdot \mathrm{S}_1^{2,1} + 2 \cdot \mathrm{k_t} \cdot \mathrm{S}_2^{2,1} \cdot \mathrm{S}_0^{2,1}$

Equation S8, $\frac{d[\mathrm{S}_0^{2,1}]}{dt} = +2 \cdot \mathrm{k_a} \cdot \mathrm{S}_0^{2,0} \cdot [\mathrm{Cu(I)L}] - \mathrm{k_a} \cdot \mathrm{S}_0^{2,1} \cdot [\mathrm{Cu(I)L}] - \mathrm{k_d} \cdot \mathrm{S}_0^{2,1} \cdot [\mathrm{XCu(II)L}] + 2 \cdot \mathrm{k_d} \cdot \mathrm{S}_0^{2,2} \cdot [\mathrm{XCu(II)L}] - \mathrm{k_t} \cdot \mathrm{S}_0^{2,1} \cdot \mathrm{S}_0^{2,1}$

Equation S9, $\frac{dS_1^{2,1}}{dt} = +2 \cdot \mathrm{k_a} \cdot \mathrm{S}_1^{2,0} \cdot [\mathrm{Cu(I)L}] - \mathrm{k_a} \cdot \mathrm{S}_1^{2,1} \cdot [\mathrm{Cu(I)L}] - \mathrm{k_d} \cdot \mathrm{S}_1^{2,1} \cdot [\mathrm{XCu(II)L}] + 2 \cdot \mathrm{k_d} \cdot \mathrm{S}_1^{2,2} \cdot [\mathrm{XCu(II)L}] + \mathrm{k_p} \cdot [\mathrm{M}] \cdot \mathrm{S}_0^{2,1} + 2 \cdot \mathrm{k_t} \cdot \mathrm{S}_1^{2,2} \cdot \mathrm{S}_0^{2,1} - \mathrm{k_t} \cdot \mathrm{S}_1^{2,1} \cdot \mathrm{S}_0^{2,1}$

Equation S10, $\frac{dS_2^{2,1}}{dt} = +2 \cdot k_\mathrm{a} \cdot \mathrm{S}_2^{2,0} \cdot [\mathrm{Cu(I)L}] - k_\mathrm{a} \cdot \mathrm{S}_2^{2,1} \cdot [\mathrm{Cu(I)L}] - k_\mathrm{d} \cdot \mathrm{S}_2^{2,1} \cdot [\mathrm{XCu(II)L}] + 2 \cdot k_\mathrm{d} \cdot \mathrm{S}_2^{2,2} \cdot [\mathrm{XCu(II)L}] + 2 \cdot k_\mathrm{p} \cdot [\mathrm{M}] \cdot \mathrm{S}_1^{2,1} + k_\mathrm{p} \cdot [\mathrm{M}] \cdot \mathrm{S}_0^{2,1} - k_\mathrm{t} \cdot \mathrm{S}_0^{2,1} \cdot \mathrm{S}_2^{2,1} + 2 \cdot k_\mathrm{t} \cdot \mathrm{S}_0^{2,1} \cdot \mathrm{S}_2^{2,2} + 4 \cdot k_\mathrm{t} \cdot \mathrm{S}_1^{2,1} \cdot \mathrm{S}_1^{2,2}$

Equation S11, $\frac{d[\mathrm{S}_0^{2,2}]}{dt} = +\mathrm{k_a} \cdot \mathrm{S}_0^{2,1} \cdot [\mathrm{Cu(I)L}] - 2 \cdot \mathrm{k_d} \cdot \mathrm{S}_0^{2,2} \cdot [\mathrm{XCu(II)L}] - \mathrm{k_t} \cdot \mathrm{S}_0^{2,2} - 2 \cdot \mathrm{k_t} \cdot \mathrm{S}_0^{2,2} \cdot \mathrm{S}_0^{2,1}$

Equation S12, $\frac{d[\mathrm{S}_1^{2,2}]}{dt} = +\mathrm{k_a} \cdot \mathrm{S}_1^{2,1} \cdot [\mathrm{Cu(I)L}] - 2 \cdot \mathrm{k_d} \cdot \mathrm{S}_1^{2,2} \cdot [\mathrm{XCu(II)L}] + 2 \cdot \mathrm{k_p} \cdot [\mathrm{M}] \cdot \mathrm{S}_0^{2,2} - \mathrm{k_t} \cdot \mathrm{S}_1^{2,2} + 4 \cdot \mathrm{k_t} \cdot \mathrm{S}_1^{2,2} \cdot \mathrm{S}_0^{2,2} - 2 \cdot \mathrm{k_t} \cdot \mathrm{S}_1^{2,2} \cdot \mathrm{S}_0^{2,1}$

Equation S13, $\frac{d[\mathrm{S}_2^{2,2}]}{dt} = +k_\mathrm{a} \cdot \mathrm{S}_2^{2,1} \cdot [\mathrm{Cu(I)L}] - 2 \cdot k_\mathrm{d} \cdot \mathrm{S}_2^{2,2} \cdot [\mathrm{XCu(II)L}] + 4 \cdot k_\mathrm{p} \cdot [\mathrm{M}] \cdot \mathrm{S}_1^{2,2} + 2 \cdot k_\mathrm{p} \cdot [\mathrm{M}] \cdot \mathrm{S}_0^{2,2} - 2 \cdot k_\mathrm{t} \cdot \mathrm{S}_2^{2,2} \cdot \mathrm{S}_0^{2,1} + 4 \cdot k_\mathrm{t} \cdot \mathrm{S}_0^{2,2} \cdot \mathrm{S}_2^{2,2} + 8 \cdot k_\mathrm{t} \cdot \mathrm{S}_1^{2,2} \cdot \mathrm{S}_1^{2,2} - k_\mathrm{t} \cdot \mathrm{S}_2^{2,2}$

Equation S14, $\frac{d[\mathrm{S}_0^{0,0}]}{dt} = +\mathrm{k_t} \cdot \mathrm{S}_0^{2,2}$

Equation S15, $\frac{d[\mathrm{S}_1^{0,0}]}{dt} = +\mathrm{k_t} \cdot \mathrm{S}_1^{2,2}$

Equation S16, $\frac{d[\mathrm{S}_2^{0,0}]}{dt} = +\mathrm{k_t} \cdot \mathrm{S}_2^{2,2}$

Equation S17, $\chi_\mathrm{rings} = \frac{S_0^{0,0}}{\sum_\sigma \sum_j S_0^{\sigma,j}}$

Where: Cu(I)L is the activator, XCu(II)L is the deactivator, RED is the reducing agent, M is the monomer, $S_0^{0,0}$ is the zero-th moment of cyclic polymers, $S_1^{0,0}$ its first moment, $S_2^{0,0}$ its second moment, $S_0^{2,0}$ is the zero-th moment of linear polymers deactivated at both chain-ends, $S_1^{2,0}$ its first moment, $S_2^{2,0}$ its second moment, $S_0^{2,1}$ is the zero-th moment of linear polymers activated at one chain-end and deactivated at the other, $S_1^{2,1}$ its first moment, $S_2^{2,1}$ its second moment, $S_0^{2,2}$ is the zero-th moment of linear polymers activated at both chain-ends, $S_1^{2,2}$ its first moment, $S_2^{2,2}$ its second moment, k_a is the kinetic rate constant of activation, k_d is the kinetic rate constant of deactivation, k_p is the kinetic rate constant of propagation, k_r is the kinetic rate constant of reduction, k_t is the kinetic rate constant of termination.

Table S1. Expression of the kinetic rate constants employed in the model.[A]

	Reaction conditions of **Table S2**, **entry**:								
$\boldsymbol{k_\mathrm{i}(\mathrm{M^{-1}min^{-1}}) = A_\mathrm{i} \cdot \exp\left(-\frac{E_i}{RT}\right)}$	**1**	**3**	**13**	**15**	**2**	**9**	**4**	**10**	*From ref:*
k_p (propagation)<br>A_p = 6.487e+8;<br>E_p = 2.925e+4	1.67 e+4	1.67 e+4	5.17 e+4	5.17 e+4	1.67 e+4	3.03 e+4	1.67 e+4	3.03 e+4	[5]
k_t (termination)<br>A_t = 8.994e+10;<br>E_t = 9.705e+3	2.70 e+9	2.70 e+9	3.93 e+9	3.93 e+9	2.70 e+9	3.29 e+9	2.70 e+9	3.29 e+9	[6,7]
k_d (deactivation)<br>A_d = 5.000e+10;<br>E_d = 2.168e+4	1.98 e+7	1.98 e+7	4.59 e+7	4.59 e+7	1.98 e+7	3.08 e+7	1.98 e+7	3.08 e+7	[8,9]
k_a (activation)<br>$A_\mathrm{a} = \boldsymbol{c_1 \cdot \phi_{\mathrm{EtOH}}}$;<br>$E_\mathrm{a} = \frac{\boldsymbol{c_2}}{\boldsymbol{\phi_{\mathrm{EtOH}}}}$	1.20 e+0	3.16 e+0	2.82 e+0	6.74 e+0	2.00 e+0	3.06 e+0	3.16 e+0	3.06 e+0	*This work*
k_r (reduction)<br>$A_\mathrm{r} = \boldsymbol{c_3 \cdot \phi_{\mathrm{EtOH}}}$;<br>$E_\mathrm{r} = \frac{\boldsymbol{c_4}}{\boldsymbol{\phi_{\mathrm{EtOH}}}}$	8.58 e+0	1.31 e+2	1.17 e+2	1.36 e+3	3.62 e+1	1.33 e+2	1.31 e+2	1.33 e+2	*This work*

A) Given: Temperature (*T*) expressed in Kelvin, ethanol fraction (ϕ_{EtOH}) rescaled such that: 0.0357 = 333 and 0.25 = 373, the optimization constants (c_1-c_4) derived as: c_1 = 1.0e+1, c_2 = 7.3 e+6, c_3 = 1.0e+9, and c_4 = 2.2e+7.

Table S2. Effect of *T* and ethanol fraction on the anomalous gelation.[A]

entry	*T* (°C)	ϕ_{EtOH} (v/v)	*time* (min)	*p*	M_n (kDa)	*Đ*	Crosslinked *or* Branched	Kinetic data / plot
**1**	60	0.0357	1740	0.72	16.0	2.06	Branched	**Table S3** / **Figure S1**
**2**	60	0.143	1080		–		Crosslinked	**Table S7** / **Figure S5**
**3**	60	0.25	1080		–		Crosslinked	**Table S4** / **Figure S2**
**4** [B]	60	0.25	1080	0.32	4.49	1.35	*neither*	**Table S9** / **Figure S7**
**5**	60	0.357	492		–		Crosslinked	
**6**	70	0.143	300		–		Crosslinked	
**7**	70	0.286	171		–		Crosslinked	
**8**	80	0.0714	1080	0.92	58.0	4.21	Branched	
**9**	80	0.143	1080		–		Crosslinked	**Table S8** / **Figure S6**
**10** [B]	80	0.143	1014	0.47	6.39	1.68	*neither*	**Table S10** / **Figure S8**
**11**	80	0.214	150		–		Crosslinked	
**12**	90	0.286	118		–		Crosslinked	
**13**	100	0.0357	540	0.92	18.5	2.25	*neither*	**Table S5** / **Figure S3**
**14**	100	0.0714	1080	0.99	61.3	7.15	Branched	
**15**	100	0.25	90	0.68	23.8	2.62	Branched	**Table S6** / **Figure S4**
**16**	100	0.357	1080	0.98	52.9	7.28	Branched	

A) Common conditions: $[\text{styrene}]_0$: $[\text{EDCP}]_0$: $[\text{CuCl}_2/\text{TPMA}]_0$: $[\text{H}_2\text{AA}]_0$: $[\text{Na}_2\text{CO}_3]_0$ = 100 : 1.06 : 0.0125 : 0.5 : 1.5 mol%, $V_{styrene}$: (V_{EtOAc} + V_{EtOH}) = 3 : 4 mL. **B)** $[\text{H}_2\text{AA}]_0$: $[\text{Na}_2\text{CO}_3]_0$ = 0.125 : 0.375 mol% with respect to styrene.

Table S3. Kinetics of **entry 1**, **Table S2** (T = 60 °C and ϕ_{EtOH} = 0.0357 v/v).[A)]

**entry**	***time* (min)**	***p***	**M_n (kDa)**	**ΔM_n (%)**	**$Đ$**
			Modeled points		
**1**	120	0.0358	–	–	–
**2**	360	0.184	3.51	+ 77.7	1.37
**3**	740	0.521	9.12	+ 72.2	1.86
**4**	875	0.595	9.71	+ 61.5	1.86
			Unmodeled points		
**5**	990	0.640	12.71	+ 96.9	1.94
**6**	1140	0.650	–	–	–
**7**	1287	0.650	12.40	+ 89.1	1.91
**8**	1480	0.674	–	–	–
**9** [B)]	1740	0.722	15.97	+ 119	2.06
**10**	1942	0.792	–	–	–

A) Common reaction conditions: [styrene]$_0$: [EDCP]$_0$: [$CuCl_2$/TPMA]$_0$: [H_2AA]$_0$: [Na_2CO_3]$_0$ = 100 : 1.06 : 0.0125 : 0.5 : 1.5 mol%, $V_{styrene}$: V_{EtOAc} : V_{EtOH} = 3 : 3.75 : 0.25 mL.

B) Branching detected by GPC Visco-MALLS.

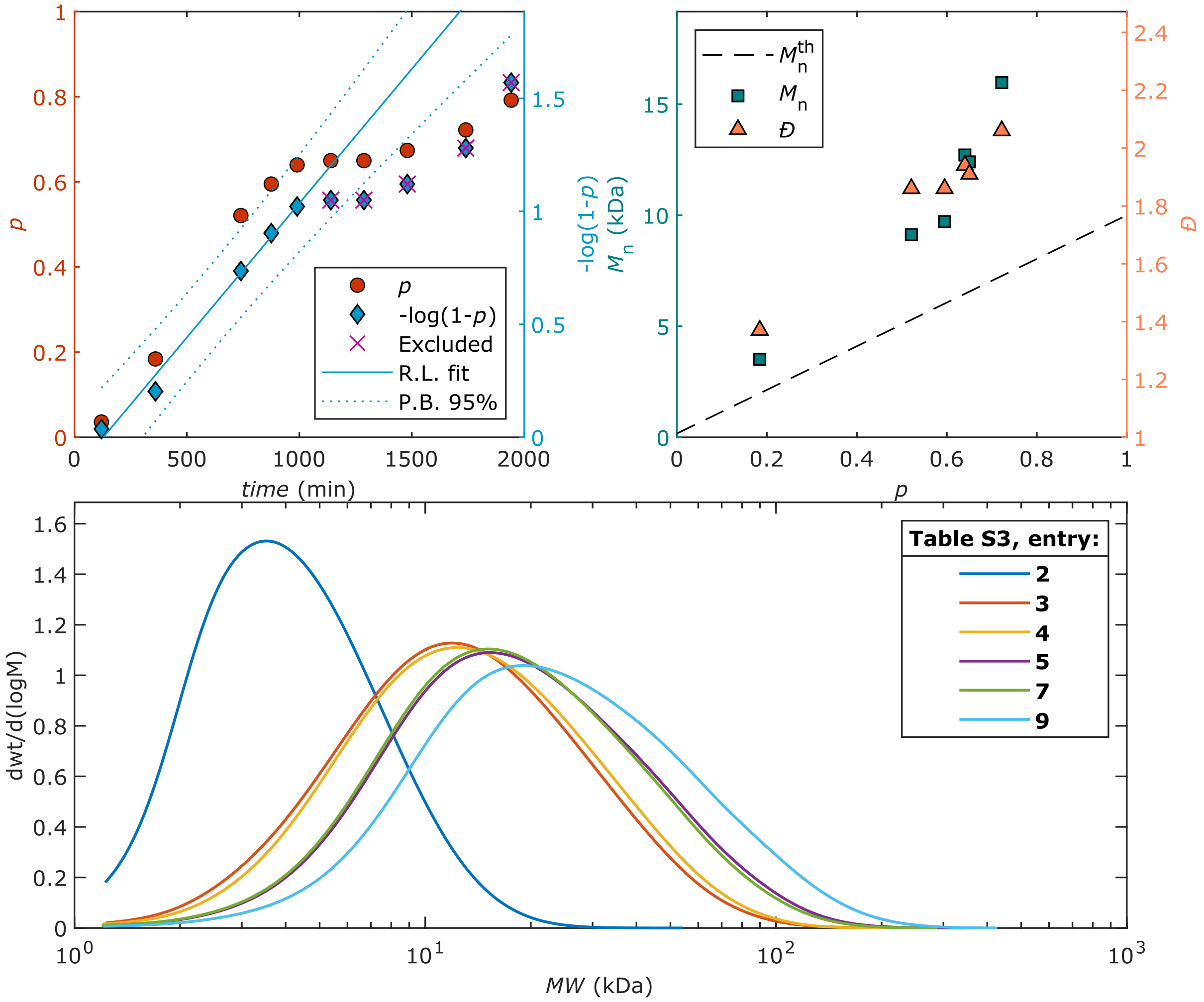

Figure S1. Graphical representation of the kinetic evolution of **entry 1**, **Table S2** (single points reported in **Table S3**). Reaction conditions: [styrene]$_0$: [EDCP]$_0$: [$CuCl_2$/TPMA]$_0$: [H_2AA]$_0$: [Na_2CO_3]$_0$ = 100 : 1.06 : 0.0125 : 0.5 : 1.5 mol%, V_{styrene} : V_{EtOAc} : V_{EtOH} = 3 : 3.75 : 0.25 mL (T = 60 °C and ϕ_{EtOH} = 0.0357 v/v). *Upper left plot*, *red circles*: *p* vs *time*; *blue diamonds*: –log(1–*p*) vs *time*. The robust bisquare linear (R.L.) fit of the first five –log(1–*p*) vs *time* points, along with the 95% prediction bounds (P.B.), is shown overlaid on the data. The fit function is –log(1–p) = P1*time + P2, with coefficients (and 95% confidence intervals): P1 = 0.001188 (0.0009461, 0.00143) and P2 = –0.152 (–0.321, 0.01704). The R-squared value is 0.9878. *Upper right plot*, *green squares*: M_n determined by GPC vs *p* against M_n^{th} vs *p* (*dashed line*); *orange triangles*: *Đ* vs *p*. *Lower plot*, molecular weight distributions.

Table S4. Kinetics of **entry 3**, **Table S2** (T = 60 °C and ϕ_{EtOH} = 0.25 v/v).[A,B]

**entry**	***time* (min)**	***p***	**M_n (kDa)**	**ΔM_n (%)**	**$Đ$**
Modeled points					
**1**	45	0.0917	3.36	+ 214	1.34
**2**	60	0.148	3.76	+ 131	1.46
**3**	75	0.199	5.75	+ 170	1.73
**4**	90	0.236	5.52	+ 122	1.74
Unmodeled points					
**5**	120	0.275	9.23	+ 221	2.11
**6**	150	0.313	–	–	–
**7**	150	0.312	11.06	+ 242	2.34
**8**	240	0.395	–	–	–
**9**	360	0.460	–	–	–

A) Common reaction conditions: [styrene]$_0$: [EDCP]$_0$: [$CuCl_2$/TPMA]$_0$: [H_2AA]$_0$: [Na_2CO_3]$_0$ = 100 : 1.06 : 0.0125 : 0.5 : 1.5 mol%, $V_{styrene}$: V_{EtOAc} : V_{EtOH} = 3 : 2.25 : 1.75 mL.

B) These reaction conditions lead to macroscopical gelation in ~7 h of reaction time.

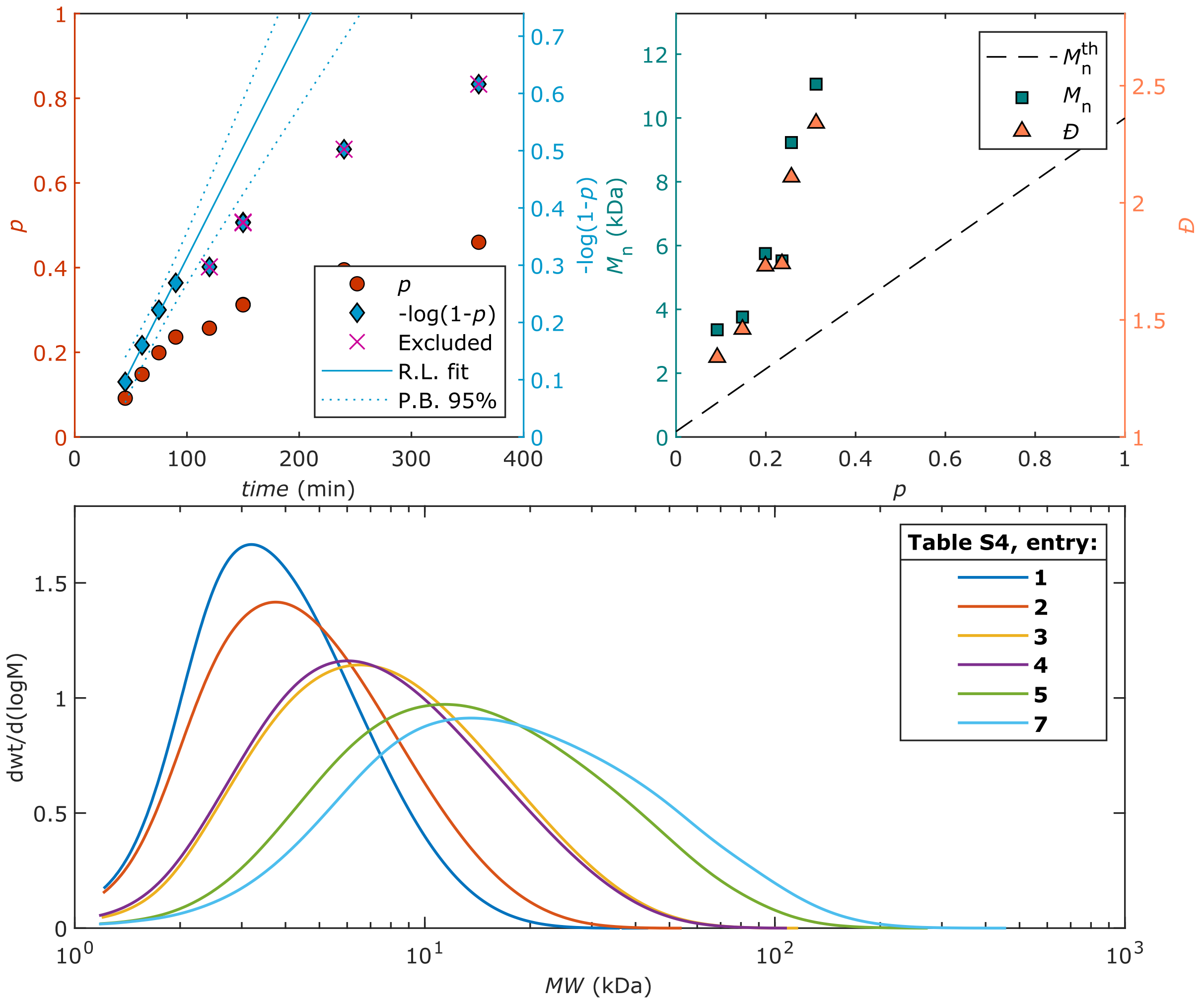

Figure S2. Graphical representation of the kinetic evolution of **entry 3**, **Table S2** (single points reported in **Table S4**). Reaction conditions: [styrene]$_0$: [EDCP]$_0$: [CuCl$_2$/TPMA]$_0$: [H$_2$AA]$_0$: [Na$_2$CO$_3$]$_0$ = 100 : 1.06 : 0.0125 : 0.5 : 1.5 mol%, V_{styrene} : V_{EtOAc} : V_{EtOH} = 3 : 2.25 : 1.75 mL (T = 60 °C and ϕ_{EtOH} = 0.25 v/v). *Upper left plot*, *red circles*: *p* vs *time*; *blue diamonds*: –log(1–*p*) vs *time*. The robust bisquare linear (R.L.) fit of the first four –log(1–*p*) vs *time* points, along with the 95% prediction bounds (P.B.), is shown overlaid on the data. The fit function is –log(1–p) = P1*time + P2, with coefficients (and 95% confidence intervals): P1 = 0.003874 (0.002968, 0.00478) and P2 = –0.07459 (–0.1376, –0.01159). The R-squared value is 0.9941. *Upper right plot*, *green squares*: M_n determined by GPC vs *p* against M_n^{th} vs *p* (*dashed line*); *orange triangles*: *Đ* vs *p*. *Lower plot*, molecular weight distributions.

Table S5. Kinetics of **entry 13**, **Table S2** (T = 100 °C and ϕ_{EtOH} = 0.0357 v/v).[A)]

entry	*time* (min)	*p*	M_n (kDa)	ΔM_n (%)	*Đ*
			Modeled points		
**1**	80	0.310	4.10	+ 27.4	1.38
**2**	100	0.397	5.04	+ 23.7	1.44
**3**	157	0.583	8.08	+ 37.0	1.55
**4**	240	0.765	–	–	–
			Unmodeled points		
**5**	271	0.794	–	–	–
**6**	331	0.836	14.78	+ 76.3	1.94
**7**	370	0.865	–	–	–
**8**	410	0.880	16.8	+ 90.3	2.16
**9**	493	0.907	–	–	–
**10**	540	0.921	18.5	+ 100	2.25

A) Common reaction conditions: [styrene]$_0$: [EDCP]$_0$: [$CuCl_2$/TPMA]$_0$: [H_2AA]$_0$: [Na_2CO_3]$_0$ = 100 : 1.06 : 0.0125 : 0.5 : 1.5 mol%, $V_{styrene}$: V_{EtOAc} : V_{EtOH} = 3 : 3.75 : 0.25 mL.

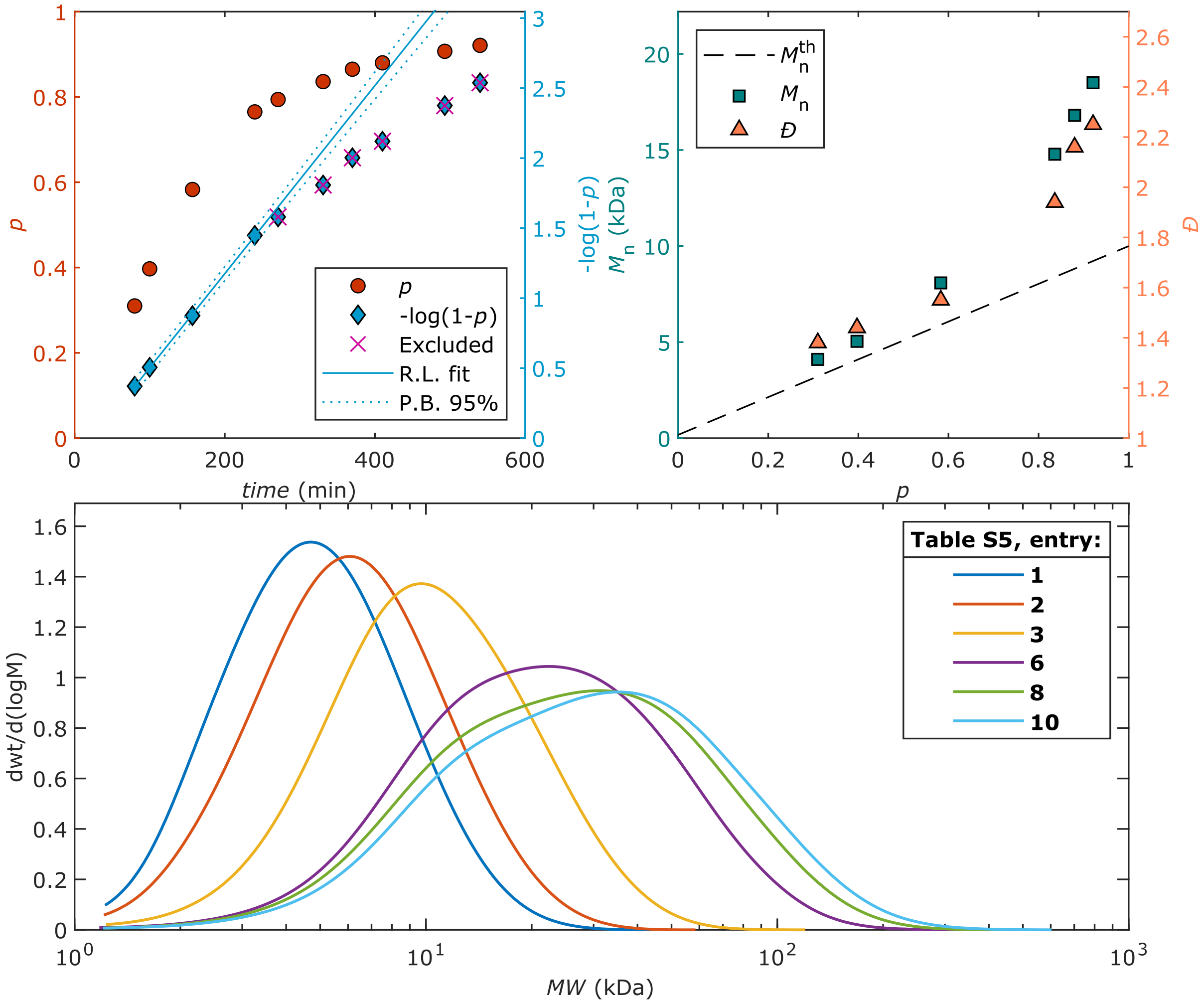

Figure S3. Graphical representation of the kinetic evolution of **entry 13**, **Table S2** (single points reported in **Table S5**). Reaction conditions: $[\text{styrene}]_0$: $[\text{EDCP}]_0$: $[\text{CuCl}_2/\text{TPMA}]_0$: $[\text{H}_2\text{AA}]_0$: $[\text{Na}_2\text{CO}_3]_0$ = 100 : 1.06 : 0.0125 : 0.5 : 1.5 mol%, V_{styrene} : V_{EtOAc} : V_{EtOH} = 3 : 3.75 : 0.25 mL (T = 100 °C and ϕ_{EtOH} = 0.0357 v/v). *Upper left plot*, *red circles*: *p* vs *time*; *blue diamonds*: –log(1–*p*) vs *time*. The robust bisquare linear (R.L.) fit of the first four –log(1–*p*) vs *time* points, along with the 95% prediction bounds (P.B.), is shown overlaid on the data. The fit function is –log(1–p) = P1*time + P2, with coefficients (and 95% confidence intervals): P1 = 0.006719 (0.006385, 0.007052) and P2 = –0.1692 (–0.2216, –0.1167). The R-squared value is 0.9997. *Upper right plot*, *green squares*: M_n determined by GPC vs *p* against M_n^{th} vs *p* (*dashed line*); *orange triangles*: *Đ* vs *p*. *Lower plot*, molecular weight distributions.

Table S6. Kinetics of **entry 15**, **Table S2** (T = 100 °C and ϕ_{EtOH} = 0.25 v/v).[A)]

**entry**	***time* (min)**	***p***	**M_n (kDa)**	**ΔM_n (%)**	**$Đ$**
			Modeled points		
**1**	30	0.127	–	–	–
**2**	50	0.421	8.30	+ 92.7	1.75
**3**	60	0.506	10.06	+ 95.7	1.81
**4**	70	0.591	17.07	+ 185	2.24
			Unmodeled points		
**5** [B)]	90	0.681	23.78	+ 246	2.62
**6** [B)]	100	0.696	28.27	+ 303	2.99
**7**	120	0.742	–	–	–
**8** [B)]	160	0.807	47.92	+ 492	6.96
**9**	180	0.828	–	–	–

A) Common reaction conditions: [styrene]$_0$: [EDCP]$_0$: [$CuCl_2$/TPMA]$_0$: [H_2AA]$_0$: [Na_2CO_3]$_0$ = 100 : 1.06 : 0.0125 : 0.5 : 1.5 mol%, $V_{styrene}$: V_{EtOAc} : V_{EtOH} = 3 : 2.25 : 1.75 mL.

B) Branching detected by GPC Visco-MALLS.

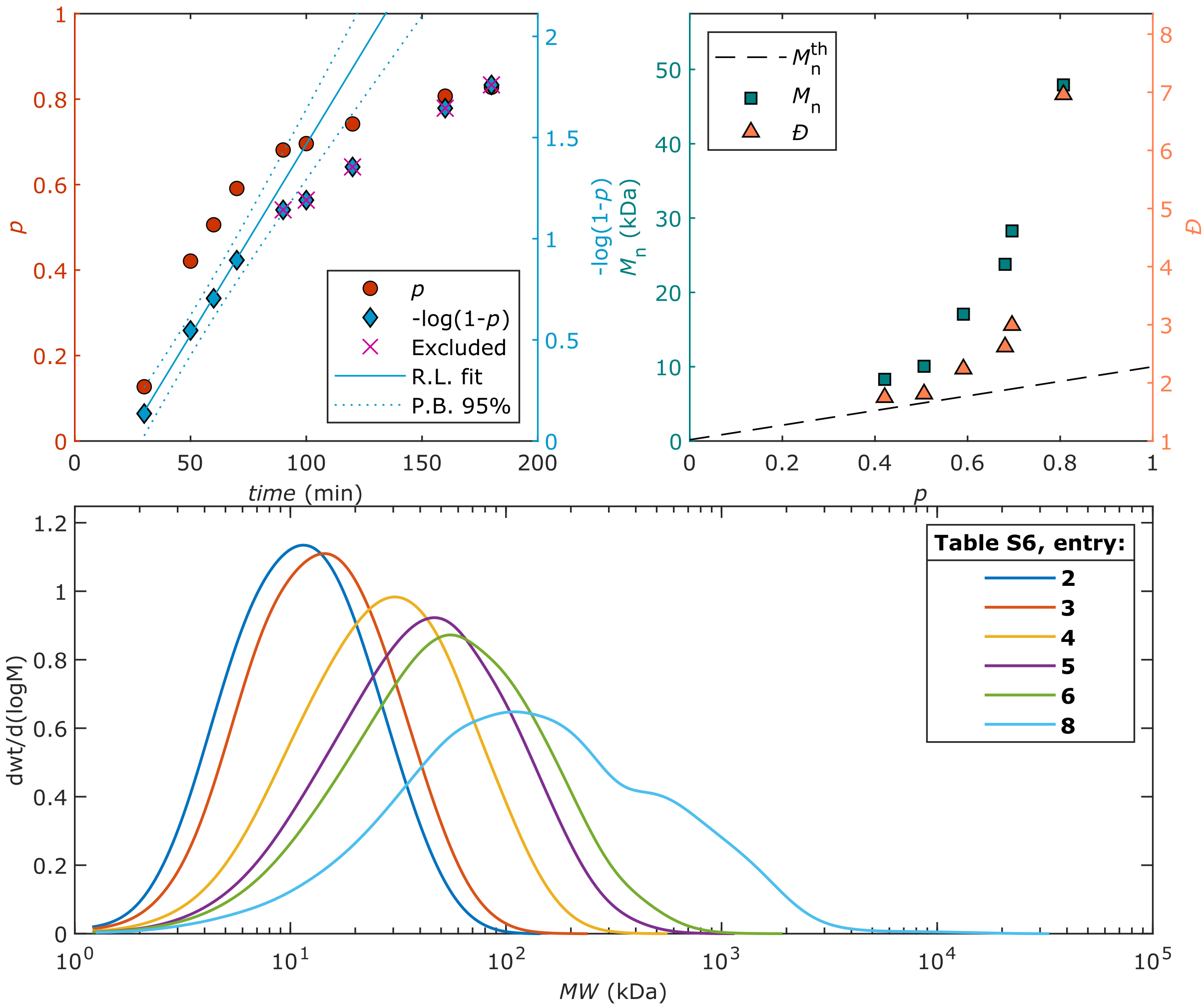

Figure S4. Graphical representation of the kinetic evolution of **entry 15**, **Table S2** (single points reported in **Table S6**). Reaction conditions: $[\text{styrene}]_0$: $[\text{EDCP}]_0$: $[\text{CuCl}_2/\text{TPMA}]_0$: $[\text{H}_2\text{AA}]_0$: $[\text{Na}_2\text{CO}_3]_0$ = 100 : 1.06 : 0.0125 : 0.5 : 1.5 mol%, V_{styrene} : V_{EtOAc} : V_{EtOH} = 3 : 2.25 : 1.75 mL (T = 100 °C and ϕ_{EtOH} = 0.25 v/v). *Upper left plot*, *red circles*: *p* vs *time*; *blue diamonds*: –log(1–*p*) vs *time*. The robust bisquare linear (R.L.) fit of the first four –log(1–*p*) vs *time* points, along with the 95% prediction bounds (P.B.), is shown overlaid on the data. The fit function is –log(1–p) = P1*time + P2, with coefficients (and 95% confidence intervals): P1 = 0.01887 (0.01587, 0.02186) and P2 = –0.4203 (–0.5837, –0.2568). The R-squared value is 0.9973. *Upper right plot*, *green squares*: M_n determined by GPC vs *p* against M_n^{th} vs *p* (*dashed line*); *orange triangles*: *Đ* vs *p*. *Lower plot*, molecular weight distributions.

Table S7. Kinetics of **entry 2**, **Table S2** (T = 60 °C and ϕ_{EtOH} = 0.143 v/v).[A)]

entry	*time* (min)	*p*	M_n (kDa)	ΔM_n (%)	*Đ*
			Unmodeled points		
**1**	85	0.129	3.55	+ 145	1.40
**2**	112	0.184	4.35	+ 119	1.50
**3**	163	0.271	6.85	+ 141	1.82
**4**	213	0.305	8.73	+ 175	1.94
**5** [B)]	288	0.387	14.49	+ 264	2.43
**6** [B)]	361	0.433	16.66	+ 275	2.59
**7** [B)]	468	0.466	19.87	+ 317	2.87
**8** [B)]	536	0.503	26.44	+ 415	3.76
**9** [B)]	672	0.519	24.63	+ 366	3.39

A) Common reaction conditions: $[styrene]_0$: $[EDCP]_0$: $[CuCl_2/TPMA]_0$: $[H_2AA]_0$: $[Na_2CO_3]_0$ = 100 : 1.06 : 0.0125 : 0.5 : 1.5 mol%, $V_{styrene}$: V_{EtOAc} : V_{EtOH} = 3 : 3 : 1 mL.

B) Branching detected by GPC Visco-MALLS.

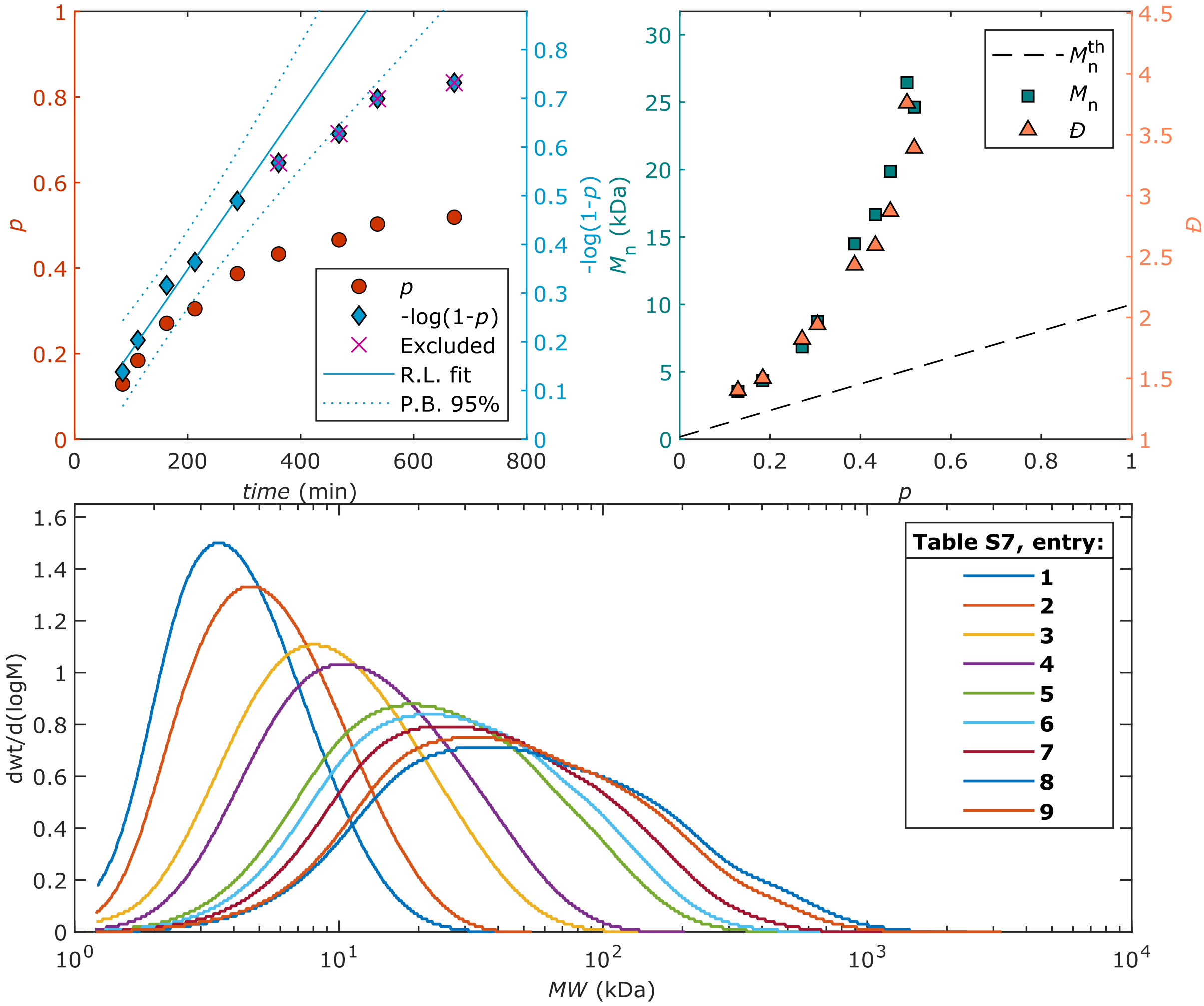

Figure S5. Graphical representation of the kinetic evolution of **entry 2**, **Table S2** (single points reported in **Table S7**). Reaction conditions: [styrene]$_0$: [EDCP]$_0$: [$CuCl_2$/TPMA]$_0$: [H_2AA]$_0$: [Na_2CO_3]$_0$ = 100 : 1.06 : 0.0125 : 0.5 : 1.5 mol%, $V_{styrene}$: V_{EtOAc} : V_{EtOH} = 3 : 3 : 1 mL (T = 60 °C and ϕ_{EtOH} = 0.143 v/v). *Upper left plot*, *red circles*: *p* vs *time*; *blue diamonds*: –log(1–*p*) vs *time*. The robust bisquare linear (R.L.) fit of the first five –log(1–*p*) vs *time* points, along with the 95% prediction bounds (P.B.), is shown overlaid on the data. The fit function is –log(1–p) = P1*time + P2, with coefficients (and 95% confidence intervals): P1 = 0.001678 (0.001234, 0.002121) and P2 = 0.01271 (–0.07023, 0.09565). The R-squared value is 0.9797. *Upper right plot*, *green squares*: M_n determined by GPC vs *p* against M_n^{th} vs *p* (*dashed line*); *orange triangles*: *Đ* vs *p*. *Lower plot*, molecular weight distributions.

Table S8. Kinetics of **entry 9**, **Table S2** (T = 80 °C and ϕ_{EtOH} = 0.143 v/v).[A)]

**entry**	***time* (min)**	***p***	**M_n (kDa)**	**ΔM_n (%)**	***Đ***
			Unmodeled points		
**1**	40	0.105	–	–	–
**2**	45	0.175	3.70	+ 95.7	1.41
**3**	60	0.287	5.93	+ 98.1	1.73
**4**	80	0.370	–	–	–
**5**	90	0.401	–	–	–
**6**	105	0.441	–	–	–
**7** [B)]	120	0.470	14.6	+ 205	2.41
**8**	135	0.506	–	–	–
**9**	150	0.534	19.87	+ 267	2.82
**10**	180	0.575	–	–	–
**11**	210	0.613	–	–	–
**12**	225	0.635	–	–	–
**13** [B)]	240	0.656	35.27	+ 433	5.57
**14**	270	0.701	40.68	+ 476	7.69

A) Common reaction conditions: $[styrene]_0$: $[EDCP]_0$: $[CuCl_2/TPMA]_0$: $[H_2AA]_0$: $[Na_2CO_3]_0$ = 100 : 1.06 : 0.0125 : 0.5 : 1.5 mol%, $V_{styrene}$: V_{EtOAc} : V_{EtOH} = 3 : 3 : 1 mL.

B) Branching detected by GPC Visco-MALLS.

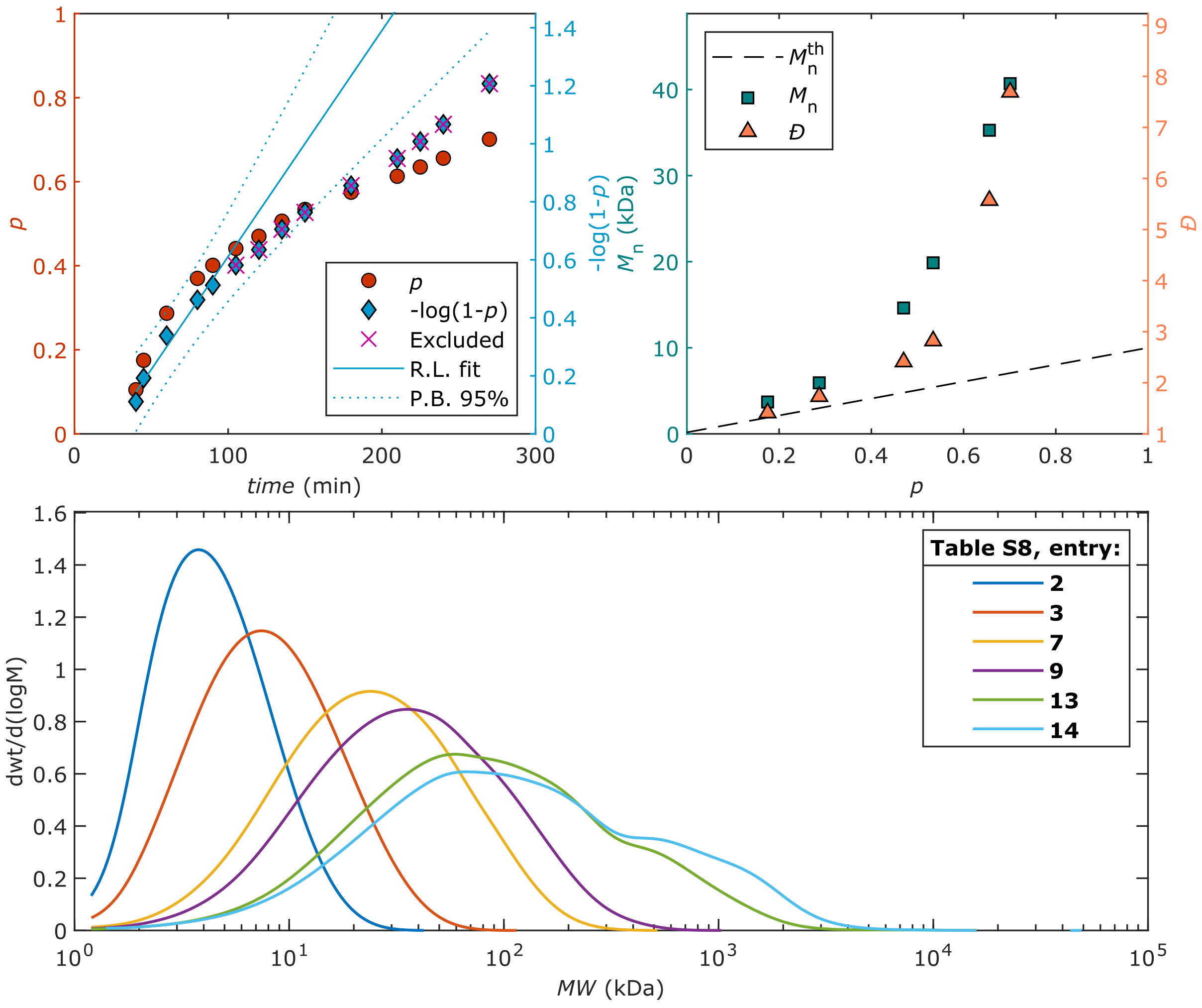

Figure S6. Graphical representation of the kinetic evolution of **entry 9**, **Table S2** (single points reported in **Table S8**). Reaction conditions: [styrene]$_0$: [EDCP]$_0$: [$CuCl_2$/TPMA]$_0$: [H_2AA]$_0$: [Na_2CO_3]$_0$ = 100 : 1.06 : 0.0125 : 0.5 : 1.5 mol%, $V_{styrene}$: V_{EtOAc} : V_{EtOH} = 3 : 3 : 1 mL (T = 80 °C and ϕ_{EtOH} = 0.143 v/v). *Upper left plot*, *red circles*: *p* vs *time*; *blue diamonds*: –log(1–*p*) vs *time*. The robust bisquare linear (R.L.) fit of the first five –log(1–*p*) vs *time* points, along with the 95% prediction bounds (P.B.), is shown overlaid on the data. The fit function is –log(1–p) = P1*time + P2, with coefficients (and 95% confidence intervals): P1 = 0.007788 (0.005213, 0.01036) and P2 = –0.1673 (–0.337, 0.002456). The R-squared value is 0.9686. *Upper right plot*, *green squares*: M_n determined by GPC vs *p* against M_n^{th} vs *p* (*dashed line*); *orange triangles*: *Đ* vs *p*. *Lower plot*, molecular weight distributions.

Table S9. Kinetics of **entry 4**, **Table S2** (T = 60 °C and ϕ_{EtOH} = 0.25 v/v).[A)]

**entry**	***time*** **(min)**	***p***	***M*$_n$ (kDa)**	**Δ*M*$_n$ (%)**	***Đ***
Unmodeled points					
**1**	180	0.038	–	–	–
**2**	330	0.113	2.81	+ 119	1.22
**3**	378	0.131	–	–	–
**4**	422	0.158	–	–	–
**5**	500	0.172	3.25	+ 74.7	1.29
**6**	553	0.185	–	–	–
**7**	570	0.209	–	–	–
**8**	610	0.202	–	–	–
**9**	720	0.246	3.77	+ 45.7	1.32
**10**	847	0.285	–	–	–
**11**	1080	0.324	4.49	+ 33.9	1.35
**12**	1440	0.403	5.33	+ 29.0	1.38
**13**	2398	0.490	6.13	+ 22.9	1.37
**14**	2945	0.485	–	–	–
**15**	4338	0.477	6.00	+ 23.5	1.36

A) Common reaction conditions: $[styrene]_0 : [EDCP]_0 : [CuCl_2/TPMA]_0 : [H_2AA]_0 : [Na_2CO_3]_0$ = 100 : 1.06 : 0.0125 : 0.125 : 0.375 mol%, $V_{styrene} : V_{EtOAc} : V_{EtOH}$ = 3 : 2.25 : 1.75 mL.

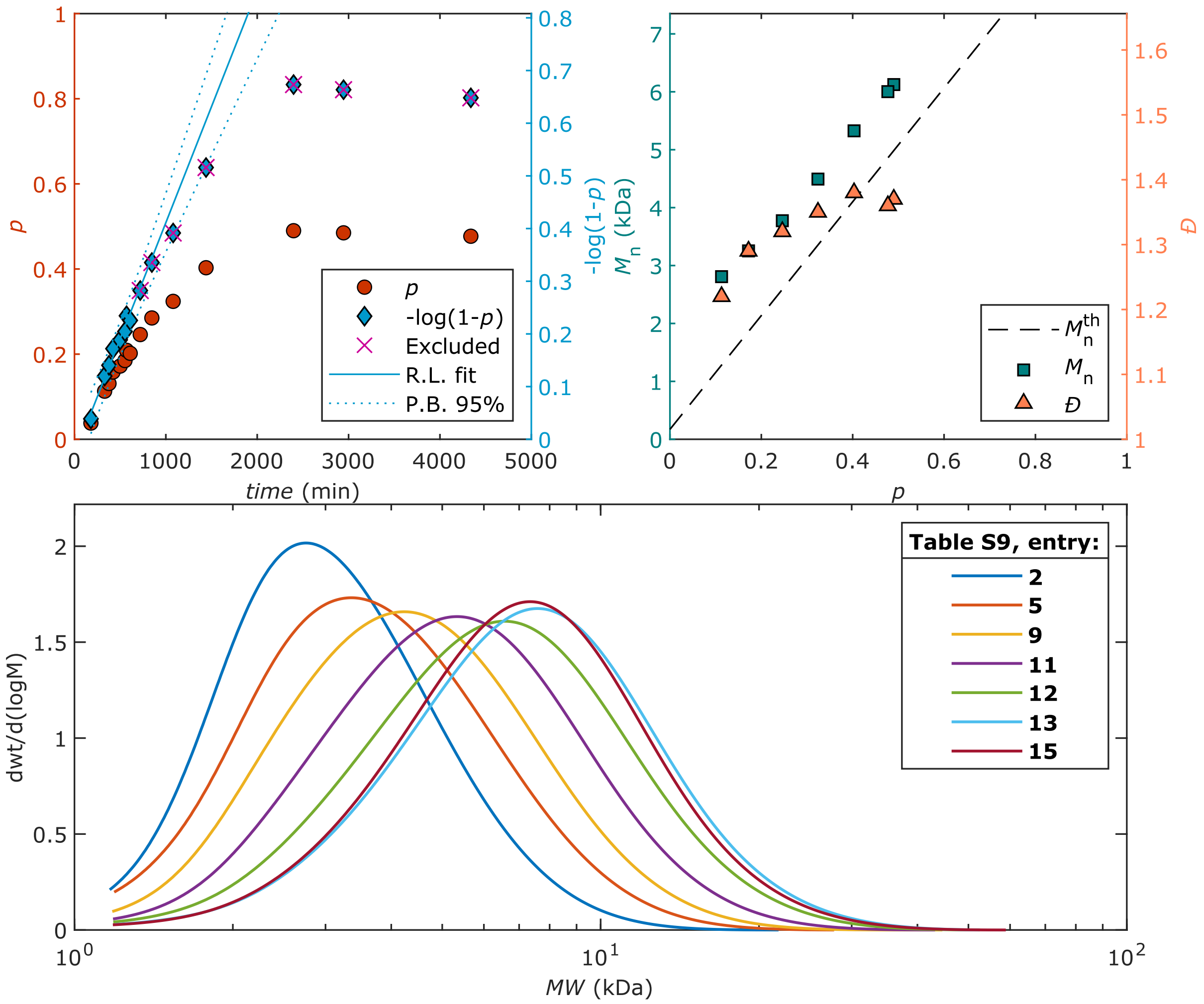

Figure S7. Graphical representation of the kinetic evolution of **entry 4**, **Table S2** (single points reported in **Table S9**). Reaction conditions: [styrene]$_0$: [EDCP]$_0$: [$CuCl_2$/TPMA]$_0$: [H_2AA]$_0$: [Na_2CO_3]$_0$ = 100 : 1.06 : 0.0125 : 0.125 : 0.375 mol%, V_{styrene} : V_{EtOAc} : V_{EtOH} = 3 : 2.25 : 1.75 mL (T = 60 °C and ϕ_{EtOH} = 0.25 v/v). *Upper left plot*, *red circles*: *p* vs *time*; *blue diamonds*: –log(1–*p*) vs *time*. The robust bisquare linear (R.L.) fit of the first eight –log(1–*p*) vs *time* points, along with the 95% prediction bounds (P.B.), is shown overlaid on the data. The fit function is –log(1–p) = P1*time + P2, with coefficients (and 95% confidence intervals): P1 = 0.0004408 (0.0003599, 0.0005216) and P2 = –0.02962 (–0.06707, 0.007828). The R-squared value is 0.9673. *Upper right plot*, *green squares*: M_n determined by GPC vs *p* against M_n^{th} vs *p* (*dashed line*); *orange triangles*: *Đ* vs *p*. *Lower plot*, molecular weight distributions.

Table S10. Kinetics of **entry 10**, **Table S2** (T = 80 °C and ϕ_{EtOH} = 0.143 v/v).[A)]

**entry**	***time* (min)**	***p***	**M_n (kDa)**	**ΔM_n (%)**	***Đ***
			Unmodeled points		
**1**	120	0.159	3.23	+ 86.4	1.31
**2**	120	0.146	–	–	–
**3**	240	0.250	4.16	+ 58.5	1.44
**4**	277	0.286	–	–	–
**5**	300	0.305	–	–	–
**6**	360	0.351	5.30	+ 46.5	1.54
**7**	430	0.394	–	–	–
**8**	480	0.418	5.83	+ 36.2	1.55
**9**	530	0.433	5.94	+ 34.4	1.51
**10**	604	0.459	–	–	–
**11**	714	0.469	–	–	–
**12**	774	0.458	6.30	+ 34.9	1.56
**13**	894	0.467	–	–	–
**14**	1014	0.473	6.39	+ 32.5	1.68

A) Common reaction conditions: [styrene]$_0$: [EDCP]$_0$: [$CuCl_2$/TPMA]$_0$: [H_2AA]$_0$: [Na_2CO_3]$_0$ = 100 : 1.06 : 0.0125 : 0.125 : 0.375 mol%, $V_{styrene}$: V_{EtOAc} : V_{EtOH} = 3 : 3 : 1 mL.

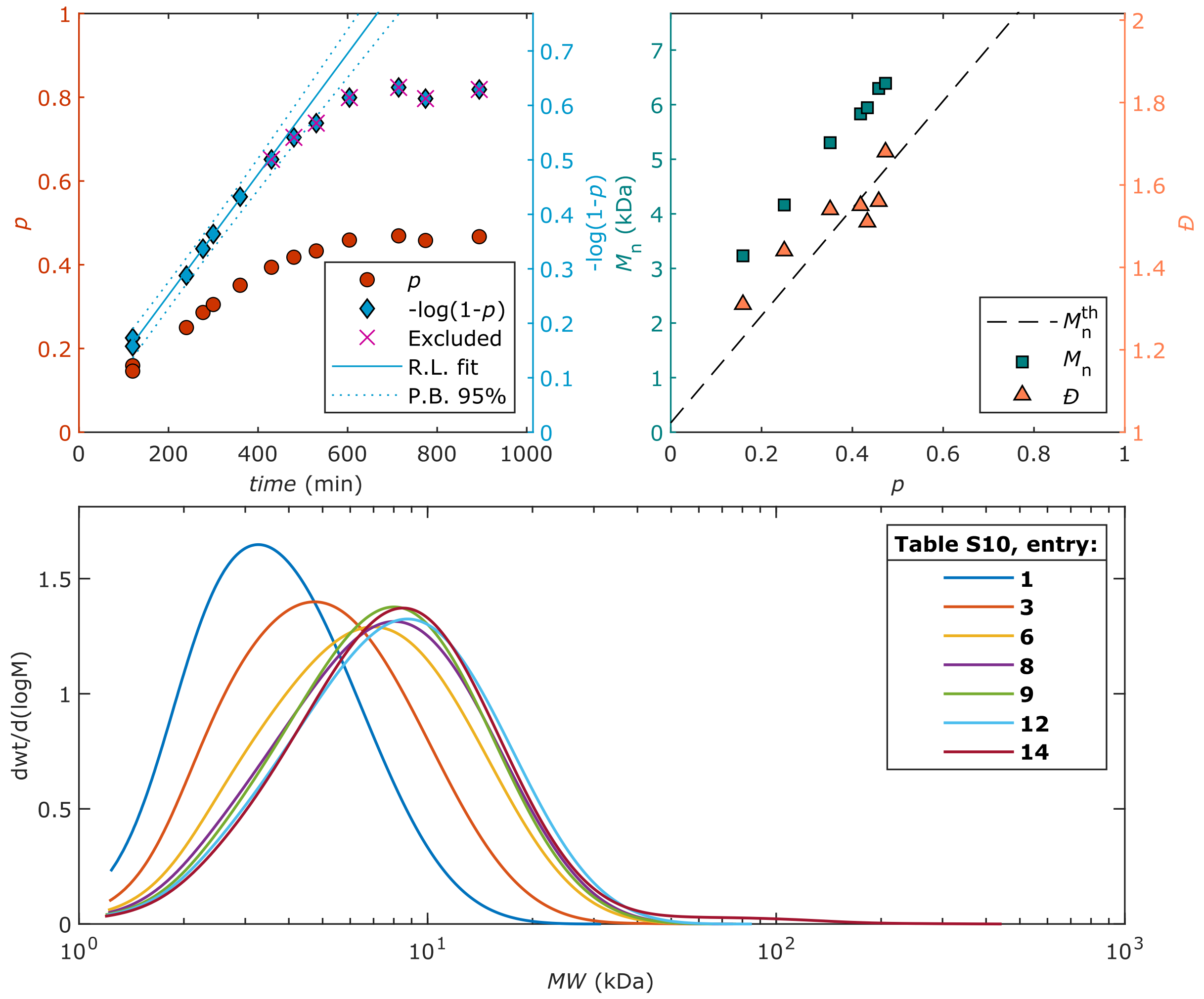

Figure S8. Graphical representation of the kinetic evolution of **entry 10**, **Table S2** (single points reported in **Table S10**). Reaction conditions: [styrene]$_0$: [EDCP]$_0$: [$CuCl_2$/TPMA]$_0$: [H_2AA]$_0$: [Na_2CO_3]$_0$ = 100 : 1.06 : 0.0125 : 0.125 : 0.375 mol%, $V_{styrene}$: V_{EtOAc} : V_{EtOH} = 3 : 3 : 1 mL (T = 80 °C and ϕ_{EtOH} = 0.143 v/v). *Upper left plot*, *red circles*: *p* vs *time*; *blue diamonds*: –log(1–*p*) vs *time*. The robust bisquare linear (R.L.) fit of the first six –log(1–*p*) vs *time* points, along with the 95% prediction bounds (P.B.), is shown overlaid on the data. The fit function is –log(1–p) = P1*time + P2, with coefficients (and 95% confidence intervals): P1 = 0.001109 (0.001008, 0.00121) and P2 = 0.02989 (0.004288, 0.05549). The R-squared value is 0.9957. *Upper right plot*, *green squares*: M_n determined by GPC vs *p* against M_n^{th} vs *p* (*dashed line*); *orange triangles*: *Đ* vs *p*. *Lower plot*, molecular weight distributions.

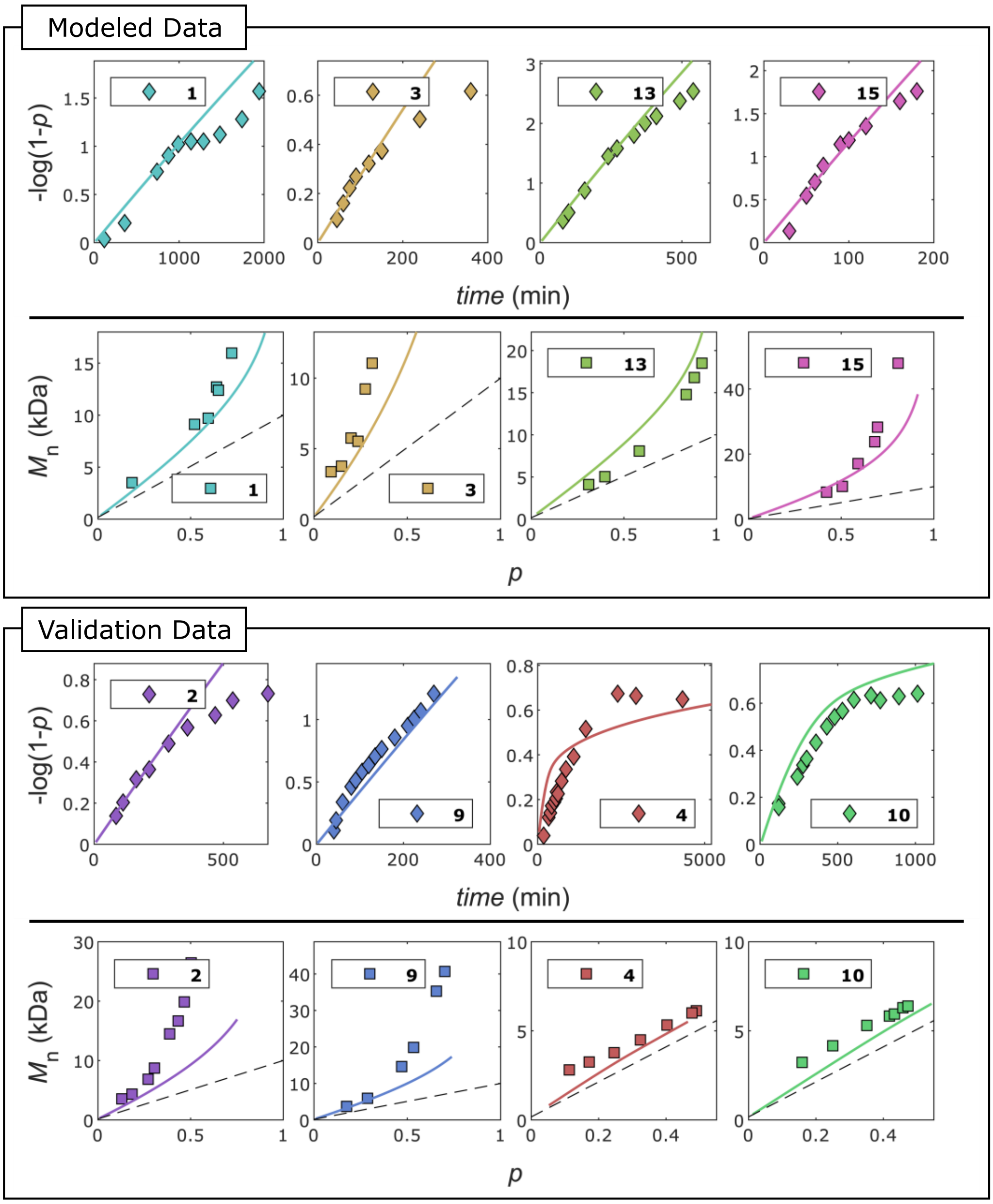

Figure S9. Kinetic evolution of selected reaction conditions reported in **Table S2**, experimental (*points*) against simulated results (*lines*). These differ by T (°C) : ϕ_{EtOH} (v/v) as follows: **1**) 60 : 0.0357, **3**) 60 : 0.25, **13**) 100 : 0.0357, **15**) 100 : 0.25, **2**) 60 : 0.143, **9**) 80 : 0.143, **4**) 60 : 0.25, **10**) 80 : 0.143. Furthermore, reactions **4** and **10** were conducted and simulated with $[H_2AA]_0$ (mol%) : $[Na_2CO_3]_0$ (mol%) = 0.125 : 0.375 instead of 0.5 : 1.5 as the others. *Upper plots*, *Modeled Data*, **entries 1**, **3**, **13**, and **15** employed to optimize the kinetic rate constants of activation and reduction. *Lower plots*, *Validation Data*, **entries 2**, **9**, **4**, and **10** employed to validate the developed model. *First and third plot*, –log(1–*p*) vs *time*. *Second and fourth plot*, M_n vs p against M_n^{th} vs p (*dashed line*).

Table S11. Influence on the gel's properties by variations in the reagents' ratio.[A)]

**entry**	**$[INI]_0$** [B)] **(n.u.)**	**$[CAT]_0$** [C)] **(n.u.)**	**$[RED]_0$** [D)] **(n.u.)**	***t_{GP}*** **(min)**	***yield*** **(mg)**	***Q*** **(v/v)**	***%G*** **(%)**
**1**	- 0.97	- 1	- 0.98	344	2334	28.9	88.3
**2**	- 0.98	- 1	+ 1	190	1806	36.4	77.4
**3**	+ 1	- 1	+ 0.97	409	2820	15.9	93.8
**4**	- 0.98	+ 1	- 1	355	2221	60.9	60.9
**5**	- 1	+ 1	- 0.36	228	1978	54.3	60.8
**6**	+ 0.18	+ 1	+ 0.97	250	2611	38.0	57.7
**7**	- 0.45	- 0.01	+ 0.30	210	2269	29.8	66.2
**8**	- 0.89	- 0.01	+ 0.78	174	1773	46.0	61.4
**9**	- 0.03	- 0.01	- 0.15	353	2919	22.7	71.4
**10**	+ 0.17	+ 1	- 0.37	534	3429	25.5	69.1

A) Common reaction conditions: $[styrene]_0$ = 100 mol%, $V_{styrene} : V_{EtOAc} : V_{EtOH} = 3 : 3 : 1$ mL, T = 70 °C, *time* = 18 h.

B) $[INI]_0$ corresponds to $[EDCP]_0$, which ranges from 1 to 2 mol% with respect to $[styrene]_0$.

C) $[CAT]_0$ corresponds to $[CuCl_2/TPMA]_0$, which ranges from 0.00625 to 0.025 mol%.

D) $[RED]_0$ corresponds to $[H_2AA]_0 : [Na_2CO_3]_0$, which range from 0.5 : 1.5 to 1 : 3 mol%. Note that $[H_2AA]_0$ and $[Na_2CO_3]_0$ are considered as a single variable, maintaining a constat ratio between the two. This is done to avoid introducing variance due to a varying significance between the two main modes of reduction that the system is capable of: reduction by HAA^- and reduction by EtO^-.[3,10]

B-D) To improve the accuracy of subsequent analysis, we have rescaled $[CAT]_0$, $[INI]_0$, and $[RED]_0$. Thus eliminating the variance caused by differences in the magnitudes of changes across the explored ranges.[11]

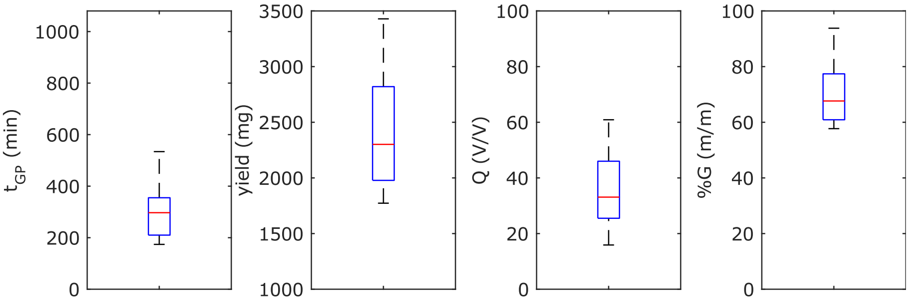

Figure S10. Boxplot of the response variables (t_{GP}, *yield*, *Q*, and *%G*) reported in **Table S11**.

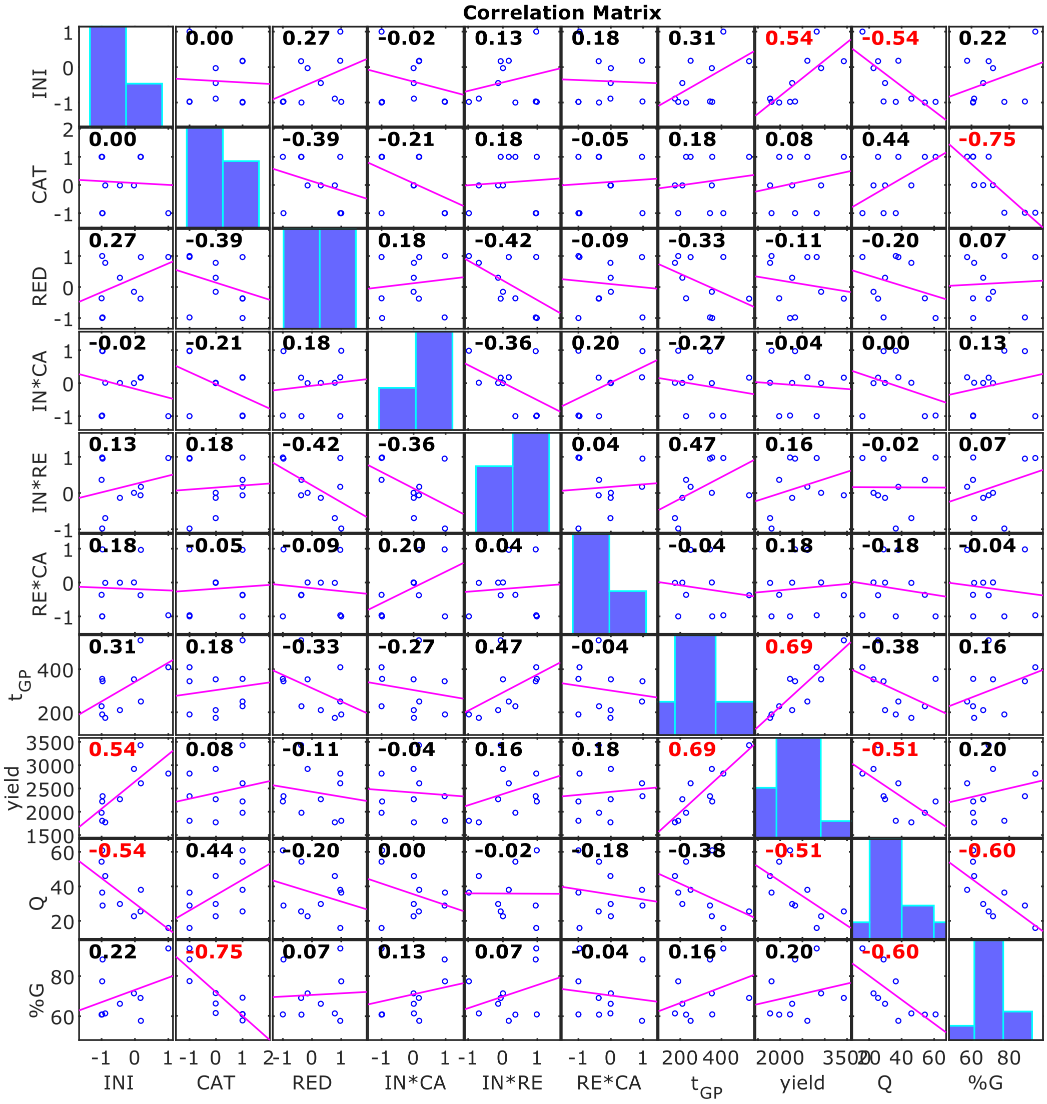

Figure S11. The correlation matrix plot of variables reported in **Table S11**, the result from the Kendall test have been superimposed on each plot.

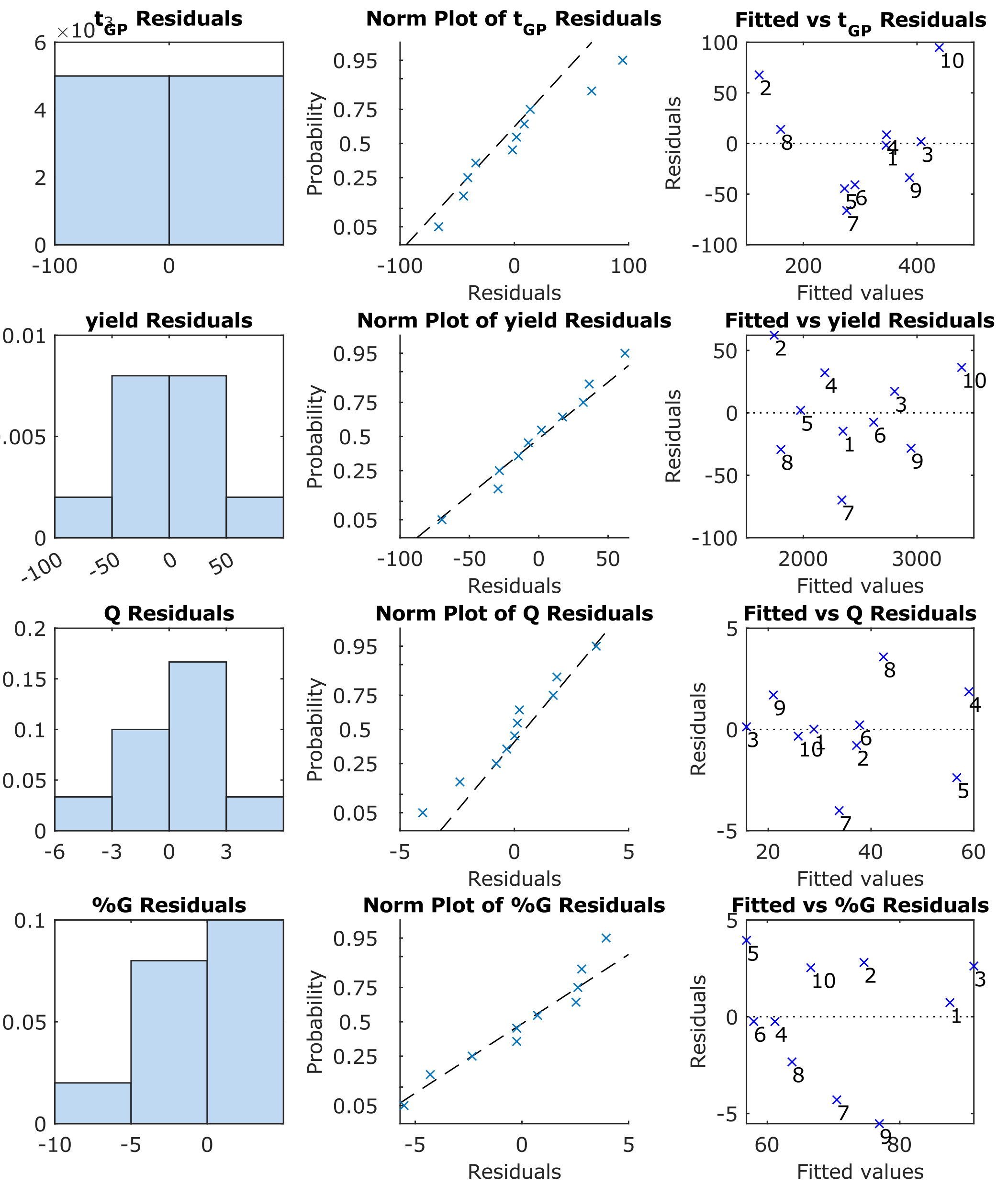

Figure S12. Diagnostic plots of the residuals for the four linear models (t_{GP}, *yield*, *Q*, and *%G*).

References

(1) Bellesia, F.; D'Anna, F.; Felluga, F.; Frenna, V.; Ghelfi, F.; Parsons, A.; Reverberi, F.; Spinelli, D. Breakthrough in the α-Perchlorination of Acyl Chlorides. *Synthesis* **2012**, *2012* (04), 605–609. https://doi.org/10.1055/s-0031-1289678.

(2) Braidi, N.; Buffagni, M.; Ghelfi, F.; Imperato, M.; Menabue, A.; Parenti, F.; Gennaro, A.; Isse, A. A.; Bedogni, E.; Bonifaci, L.; Cavalca, G.; Ferrando, A.; Longo, A.; Morandini, I. Copper-Catalysed "Activators Regenerated by Electron Transfer" "Atom Transfer Radical Polymerisation" of Styrene from a Bifunctional Initiator in Ethyl Acetate/Ethanol, Using Ascorbic Acid/Sodium Carbonate as Reducing System. *Macromol. Res.* **2020**, *28* (8), 751–761. https://doi.org/10.1007/s13233-020-8091-3.

(3) Braidi, N.; Parenti, F.; Scurani, G.; Tassinari, F.; Buffagni, M.; Bonifaci, L.; Cavalca, G.; Pettenuzzo, N.; Ghelfi, F. Influences of Nitrogen Base Excess on ARGET ATRP of Styrene with Ascorbic Acid Acetonide and Traces of Oxygen and Water. *Polym. Chem.* **2023**, *14* (13), 1567–1576. https://doi.org/10.1039/D2PY01373H.

(4) Mastan, E.; Zhu, S. Method of Moments: A Versatile Tool for Deterministic Modeling of Polymerization Kinetics. *Eur. Polym. J.* **2015**, *68,* 139–160. https://doi.org/10.1016/j.eurpolymj.2015.04.018.

(5) Deady, M.; Mau, A. W. H.; Moad, G.; Spurling, T. H. Evaluation of the Kinetic Parameters for Styrene Polymerization and Their Chain Length Dependence by Kinetic Simulation and Pulsed Laser Photolysis. *Makromol. Chem.* **1993**, *194* (6), 1691–1705. https://doi.org/10.1002/macp.1993.021940617.

(6) Mahabadi, H. K.; O'Driscoll, K. F. Absolute Rate Constants in Free-Radical Polymerization. III. Determination of Propagation and Termination Rate Constants for Styrene and Methyl Methacrylate. *J. Macromol. Sci. Part - Chem.* **1977**, *11* (5), 967–976. https://doi.org/10.1080/00222337708061301.

(7) Matheson, M. S.; Auer, E. E.; Bevilacqua, E. B.; Hart, E. J. Rate Constants in Free Radical Polymerization. III. Styrene [1]. *J. Am. Chem. Soc.* **1951**, *73* (4), 1700–1706. https://doi.org/10.1021/ja01148a082.

(8) Soerensen, N.; Barth, J.; Buback, M.; Morick, J.; Schroeder, H.; Matyjaszewski, K. SP-PLP-EPR Measurement of ATRP Deactivation Rate. *Macromolecules* **2012**, *45* (9), 3797–3801. https://doi.org/10.1021/ma300420j.

(9) Tang, W.; Kwak, Y.; Braunecker, W.; Tsarevsky, N. V.; Coote, M. L.; Matyjaszewski, K. Understanding Atom Transfer Radical Polymerization: Effect of Ligand and Initiator Structures on the Equilibrium Constants. *J. Am. Chem. Soc.* **2008**, *130* (32), 10702–10713. https://doi.org/10.1021/ja802290a.

(10) Braidi, N.; Buffagni, M.; Ghelfi, F.; Parenti, F.; Gennaro, A.; Isse, A. A.; Bedogni, E.; Bonifaci, L.; Cavalca, G.; Ferrando, A.; Longo, A.; Morandini, I. ARGET ATRP of Styrene in EtOAc/EtOH Using Only Na2CO3 to Promote the Copper Catalyst Regeneration. *J. Macromol. Sci. Part Pure Appl. Chem.* **2021**, *58* (6), 376–386. https://doi.org/10.1080/10601325.2020.1866434.

(11) Singh, D.; Singh, B. Investigating the Impact of Data Normalization on Classification Performance. *Appl. Soft Comput.* **2020**, *97*, 105524. https://doi.org/10.1016/j.asoc.2019.105524.
